# Dynamic insights into the effects of nonsynonymous polymorphisms (nsSNPs) on loss of TREM2 function

**DOI:** 10.1038/s41598-022-13120-5

**Published:** 2022-06-07

**Authors:** Raju Dash, Yeasmin Akter Munni, Sarmistha Mitra, Ho Jin Choi, Sultana Israt Jahan, Apusi Chowdhury, Tae Jung Jang, Il Soo Moon

**Affiliations:** 1 Department of Anatomy, Dongguk University College of Medicine, Gyeongju, 38066 Republic of Korea; 2 Department of Biotechnology and Genetic Engineering, Noakhali Science and Technology University, Noakhali, 3814 Bangladesh; 3 Department of Pharmaceutical Science, North-South University, Dhaka, 1229 Bangladesh; 4 Department of Pathology, Dongguk University College of Medicine, Gyeongju, 38066 Republic of Korea

**Keywords:** Genome informatics, Protein analysis, Protein folding, Protein function predictions, Protein structure predictions, Proteome informatics, Computational biophysics, Disease genetics, Molecular modelling, Neurological disorders

## Abstract

Single nucleotide variations in Triggering Receptor Expressed on Myeloid Cells 2 (TREM2) are associated with many neurodegenerative diseases, including Nasu-Hakola disease (NHD), frontotemporal dementia (FTD), and late-onset Alzheimer's disease because they disrupt ligand binding to the extracellular domain of TREM2. However, the effects of nonsynonymous single nucleotide polymorphisms (nsSNPs) in TREM2 on disease progression remain unknown. In this study, we identified several high-risk nsSNPs in the *TREM2* gene using various deleterious SNP predicting algorithms and analyzed their destabilizing effects on the ligand recognizing region of the TREM2 immunoglobulin (Ig) domain by molecular dynamics (MD) simulation. Cumulative prediction by all tools employed suggested the three most deleterious nsSNPs involved in loss of TREM2 function are rs549402254 (W50S), rs749358844 (R52C), and rs1409131974 (D104G). MD simulation showed that these three variants cause substantial structural alterations and conformational remodeling of the apical loops of the TREM2 Ig domain, which is responsible for ligand recognition. Detailed analysis revealed that these variants substantially increased distances between apical loops and induced conformation remodeling by changing inter-loop nonbonded contacts. Moreover, all nsSNPs changed the electrostatic potentials near the putative ligand-interacting region (PLIR), which suggested they might reduce specificity or loss of binding affinity for TREM2 ligands. Overall, this study identifies three potential high-risk nsSNPs in the *TREM2* gene. We propose further studies on the molecular mechanisms responsible for loss of TREM2 function and the associations between TREM2 nsSNPs and neurodegenerative diseases.

## Introduction

Microglial cells in brain express an innate immune cell surface receptor called Triggering Receptor Expressed in Myeloid cells 2 (TREM2), which controls a wide range of microglial immune functions, including chemotaxis, phagocytosis, autophagy, survival and proliferation, proinflammatory cytokine production, and lipid metabolism^1–7^. TREM2 is activated when its ligand-binding domain interacts with a broad range of putative ligands, such as oligomeric amyloid-β^8^, bacterial lipopolysaccharides, lipidated apolipoproteins like ApoA, ApoE, and CLU^9^, various sphingomyelin and phospholipids^9,10^, or nucleic acids^11^, though it shows a preference for anionic substrates^12,13^. Ligand binding initiates downstream signaling via the recruitments of co-receptor DNAX-activation protein 12 (DAP12) and SYK, ERK, PLCG2, or NFAT^14,15^. In cerebrospinal fluid (CSF), TREM2 is also detected in a soluble form (sTREM2)^9^, which is produced by the proteolytic actions of ADAM10 and ADAM17^16,17^ or alternative transcription^18^. TREM2 plays a critical neuroprotective during early and mid-term Alzheimer's disease (AD), as it suppresses the Aβ diffusion and accumulation by regulating microglial activation around amyloid plaques^3,19,20^. Furthermore, TREM2 overexpression is also associated with the clearance of soluble and insoluble Aβ42 aggregates^21,22^. Studies suggest sTREM2 physically binds to Aβ42 and inhibits its polymerization and have reported that sTREM2 overexpression ameliorates AD in a mouse model^23^ and protects cognitive reserve^24^.

Current genome-wide association studies indicate loss of TREM2 function due to the presence of homozygous or heterozygous variants is highly associated with the progression of many neurodegenerative disorders, including AD, Nasu-Hakola disease (NHD), Frontotemporal dementia (FTD), and Parkinson's disease (PD)^25–29^. Molecular modeling of the TREM2 ectodomain showed that AD-associated variants locate on the extracellular surface near the putative ligand-interacting region (PLIR), while NHD-associated variants are grossly damaging by frameshift, truncation, or unfolding. The most common variants associated with AD risk are R47H and R62H^25,26^, which reportedly disrupt ligand interaction but do not markedly affect protein structure or stability^13,30,31^. Dynamics studies conducted by molecular simulation showed that AD-associated variants induced flexibility in the three loops of the complementarity-determining region (CDR) adjacent to the PLIR, which is responsible for ligand binding and recognition. This flexibility ultimately disrupts the structural integrity of the core structure of the Ig domain and between these CDRs, and results in the exposure of negatively charged buried residues^32^. These structural consequences are most severe when the structure contains FTD and NHD mutations and are associated with complete loss of function^7,33–35^. Other studies suggest that TREM2 variants at Q33X and W191X might influence loss of TREM2 function^28,36^. Although many biophysical and molecular dynamics-based studies have characterized the impacts of several known risk-associated variants^13,15,31,32^, the disease triggering potentiality of TREM2 nonsynonymous single nucleotide polymorphisms (nsSNPs) remains uncharacterized.

In silico methods provide an effective means of finding deleterious nsSNPs in specific genes^15,37–40^. Thus, we utilized bioinformatic prediction-based tools to identify high-risk SNPs and then all-atom MD simulation to analyze the magnitudes of their effects on TREM2 structure. The MD simulation findings obtained identified three high-risk nsSNPs that alter TREM2 Ig domain structure and destabilize ligand-binding regions and concurred with previous reports^15,31,32^.

## Results 

### Identification of deleterious nsSNPs in *TREM2*

**Figure 1:**
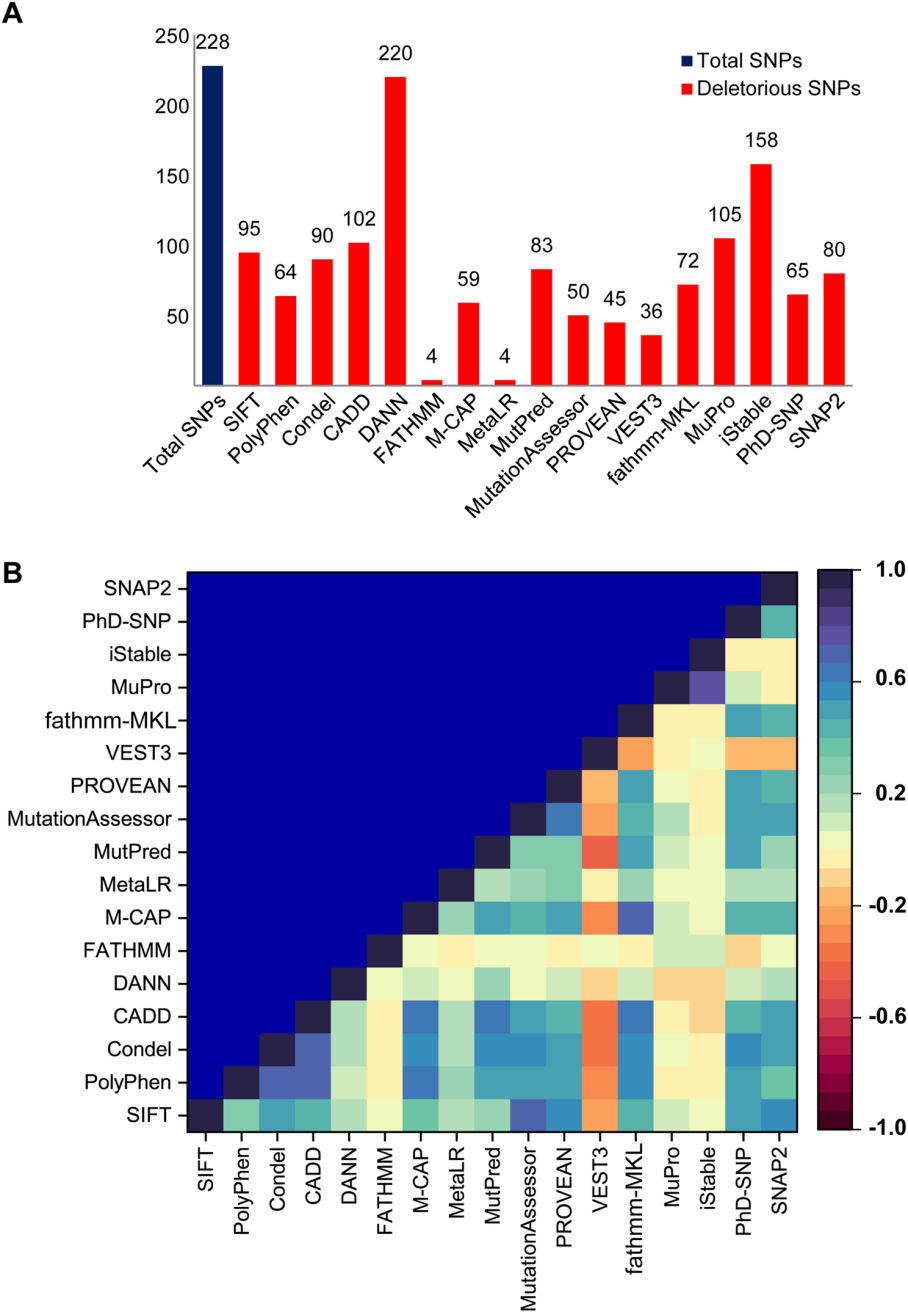
Identification of deleterious SNPs in *TREM2* using different computational algorithms. (**A**) Numbers of total and deleterious SNPs predicted by each tool. (**B**) Pairwise correlations between deleterious SNP predicting algorithms presented as a color-coded heatmap, which shows a positive correlation ( +) when algorithmic predictions agreed and a negative correlation (−) when they disagreed.

Missense variants play vital roles in many complex diseases by modulating in vivo protein functions^41,42^. Available missense SNPs in the dbSNP database (a total of 228 SNPs) were subjected to analysis to determine their effects on TREM2 structural stability and dynamics (Supplementary file 1). Using structure and sequence-based approaches (Table S1, Supplementary file 2), a total of 17 in silico nsSNP prediction algorithms were used to analyze these SNPs. In silico nsSNP prediction algorithms such as SIFT, PolyPhen, Condel, CADD, DANN, FATHMM, M-CAP, MetaLR, MutPred, MutationAssessor, PROVEAN, VEST3, fathmm-MKL, MuPro, iStable, PhD-SNP, and SNAP2 were used in this study (Table S1). The DANN algorithm predicted the highest number of deleterious SNPs, while FATHMM and MetaLR predicted the lowest number (Fig. 1A). The predictions of all algorithms were found to correlate significantly with one another. However, two algorithms, FATHMM and VEST3, produced negative correlations with other tools (Fig. 1B). SNPs that have been identified as deleterious by the most of the tools are often more likely to be deleterious than other SNPs ^43,44^. Three SNPs rs549402254 (W50S), rs749358844 (R52C), and rs1409131974 (D104G) were predicted by all algorithms, that is, by at least 15 tools, were considered high-risk nsSNPs and subjected to further analysis (Table S2, Supplementary file 2).

## Structural consequences by molecular dynamics (MD) simulation

**Figure 2:**
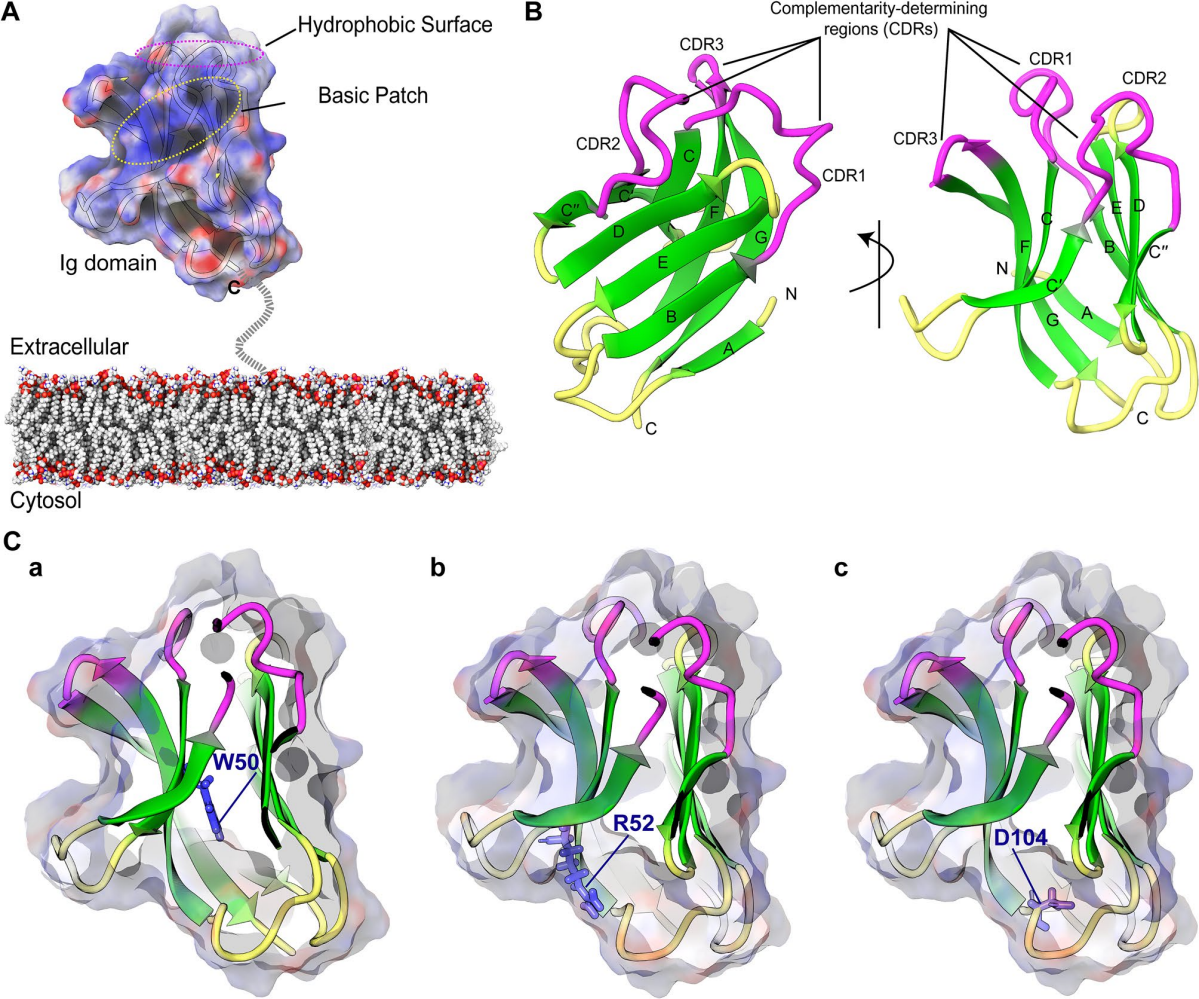
Molecular architecture showing the domain organization of TREM2 and the locations of SNPs. (**A**) In the cell membrane, TREM2 presents its immunoglobulin (Ig) domain (ectodomain) in surface view mode. The putative ligand-interacting region (PLIR) is indicated by the dotted yellow circle. (**B**) Three-dimensional representation of TREM2 Ig domain, CDRs are highlighted in pink and labeled with β-strands. (**C**) Locations of the identified SNPs in the 3-D structure of wild-type TREM2 Ig, where W50S (**a**), R52C (**b**), and D104G (**c**) are represented by blue sticks.

MD simulations were performed for variants and wild-type structures to obtain detailed structural and dynamic insights of the roles played by the identified nsSNPs in *TREM2*. Previous biophysical studies revealed that the TREM2 Ig domain consists of a hydrophobic patch and contributes to ligand binding (Fig. 2A). This region, which is also known as the complementarity-determining region (CDR), is composed of three major loops (CDR1 to CDR3) (Fig. 2B). A putative ligand-interacting region (PLIR) is also located in the Ig domain and displays a positively charged patch of surface-exposed residues that includes residues from the CDR2 and βC″ strands. Figure 2 shows that the variants W50S and R52C are located near the PLIR in the βC strand (Fig. 2Ca&b) and are buried, whereas D104G is located in the loop between βE and βF (Fig. 2Cc).

MD simulation was used to analyze the structural stabilities and dynamics of the three TREM2 variants. As described in “Methods”, three independent simulations were run for each variant and wild-type for 500 ns (a total of 1.5 μs) and subjected to root mean square deviation (RMSD) analysis to determine equilibrated trajectories by considering initial protein backbone structures (Supplemental Video 1, 2, 3 & 4). As shown in Figure S1 (Supplementary file 2), all variants and wild-type achieved equilibration after around ~ 100 ns of simulation and remained stable thereafter with a maximum RMSD of < 3.0 Å. To improve conformational sampling efficiency in the simulated trajectory analysis, the last 300 ns of trajectory from each run was extracted and concatenated to a sub-trajectory of 900 ns for further analysis. To confirm the sufficiency of conformational sampling, we analyzed sub-trajectory cosine contents, which have been shown to be good indicators of sufficient trajectory sampling to achieve convergence^45–48^. When the cosine content value is high (i.e., close to 1), protein dynamics resemble random diffusion, indicating inadequate sampling. In contrast, a cosine content near 0 indicates convergent sampling. Cosine content analysis showed that all trajectories had cosine values < 0.1 (Table S3, Supplementary file 2)^47^, which indicated trajectories were convergent and appropriate for detailed analysis^49^.

### Alterations in conformational stabilities

To examine the variations in the conformational stabilities of variant structures, RMSD values of the equilibrated trajectories, which indicate geometrical similarities between structures, were calculated for TREM2 variants and the wild-type^50,51^. As shown in Figure S2A (Supplementary file 2), TREM2 W50S, R52C, and D104G variants (Figures S2Aa, b & c, Supplementary file 2) had larger RMSD deviations than the wild-type (Figure S2dAd, Supplementary file 2), and D104G exhibited a large RMSD distribution in the density plot shifted right of wild-type distribution (Figures S2Ac, Supplementary file 2) with a maximum at ~ 1.4 Å. To confirm these deviation changes, we calculated radius of gyration (Rg), as they are indicators of protein compactness and lack of flexibility (Figures S2B Supplementary file 2). Rg analysis showed that TREM2 W50S, R52C, and D104G variants had greater Rg values than the wild-type (Figures S2Ba,b&c, Supplementary file 2), and the difference was marked for R52C but slight for W50S and D104G. Since all variants had higher Rg values than the wild-type, we calculated solvent accessible surface areas (SASAs) (Figure S2C, Supplementary file 2), which reflect surface areas accessible for biomolecular interactions. Figures S2Ca,b&c (Supplementary file 2) show that the TREM2 structure containing all three variants had a greater SASA distribution than the wild-type (> 64 nm^2^) (Figure S2Cd, Supplementary file 2). D104G had a higher average SASA than W50S or R52C and exhibited a wider SASA distribution (64 to 67 nm^2^) (Figure S2Cc, Supplementary file 2). RMSD and Rg calculations and SASA analysis indicated all variants exhibited conformational changes in the structure of TREM2, which suggested substantial domain or regional fluctuations. Moreover, Rg and SASA calculations collectively suggested that the presence of variants in TREM2 reduces structural compactness, and thus, increases protein flexibility.

### Changes in regional flexibility and correlative motion

**Figure 3:**
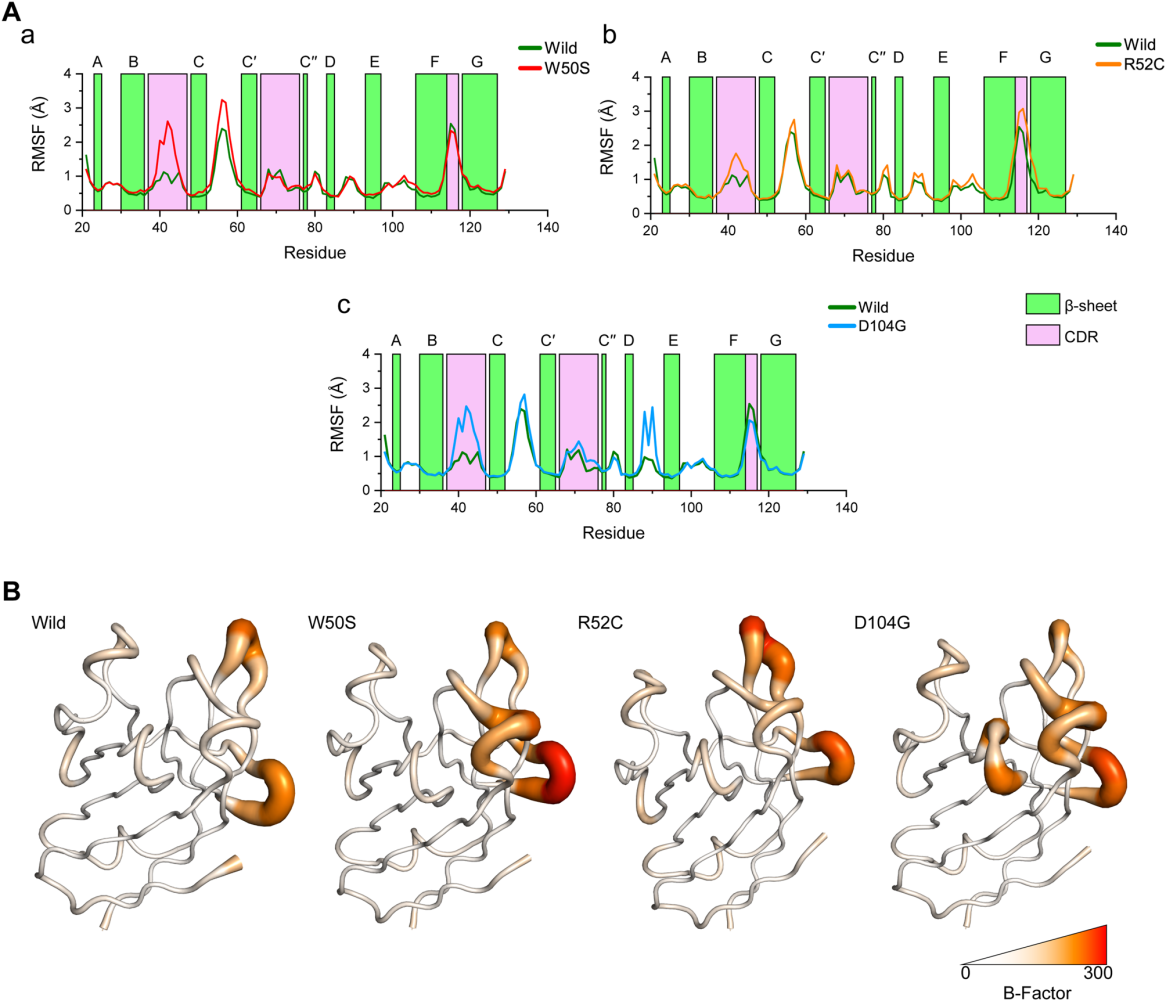
Changes in residual flexibilities during simulation. (**A**) Changes are represented by root mean square fluctuation (RMSF) plots for W50S (**a**), R52C (**b**), and D104G (**c**) versus the wild-type structure. Color bars represents β-sheet propensity and CDRs. (**B**) Comparison of TREM2 Ig domain fluctuations as demonstrated by B-factor coloring and thickness. RMSF values were converted to B-factors to represent structures in colored putty representations . Coloring was made by white-red spectrum, white means B-factor with a value 0, while red means 300. Tube thicknesses also indicate levels of fluctuation, i.e., tube thicknesses represent levels of fluctuation.

Root mean square fluctuation (RMSF) analysis was used to quantify TREM residual flexibility in the equilibrium state and identify regions that most influence conformational motion and stability^52^. Results are plotted in Fig. 3, which shows D104G increased residual fluctuation as compared with the wild-type (Fig. 3Ac) and that these effects were more pronounced in the CDR1 loop and the βE region. W50S and R52C also increased fluctuation in the CDR1 loop to a level higher than that of wild-type (Fig. 3Ab). W50S also showed substantially greater fluctuations in residues of the βC-βC' loop than the wild-type (Fig. 3B). These observations suggest that the identified variants might interrupt intramolecular networks essential for the stability of functional regions, like apical CDRs.

**Figure 4:**
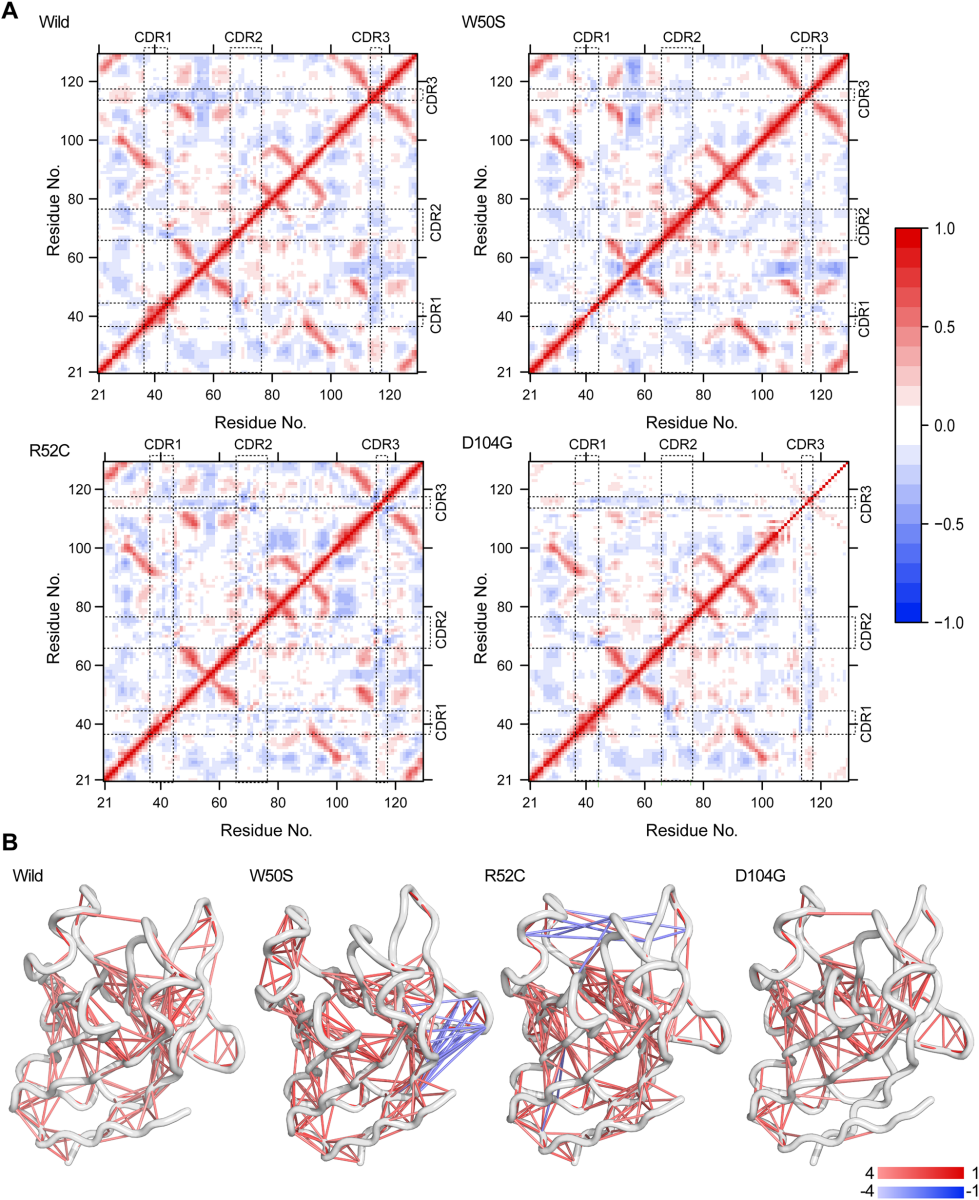
Alterations in the correlated motions in TREM2 variants. (**A**) The dynamic cross-correlation matrix (DCCM) plot shows anticorrelated and correlated motions between every two residues in the structure. Here a perfect correlated motion is denoted by red (+ 1), and an anticorrelated motion by blue (− 1). CDRs are indicated by dotted boxes. (**B**) DCCM representation in the structural view of TREM2, where lines denote correlations between two residues. A blue line indicates anticorrelated motions, and a red line denotes positive correlations. Color intensities indicate strengths of correlations.

Dynamic cross-correlation maps (DCCMs) were constructed based on equilibrated trajectories to investigate pairwise relative motion amid all residue pairs in the TREM2 Ig domain and enable correlations between these regional changes (Fig. 3) and dynamic motions as determined using color-coded maps. In accord with RMSF analysis, DCCM also revealed notable changes between the variants and the wild-type, and a substantial reduction in relative motion was observed for D104G (Fig. 4A). W50S increased anticorrelated motion between the βC-βC' loop and the N-terminal segment of TREM2 (residues, 100 to 129), and this loop (βC-βC') also exhibited high flexibility by RMSF analysis. In addition, all CDR loops in R52C showed slight increases in anticorrelated motions relative to each other (Fig. 4B). On the other hand, correlative motion was substantially reduced in the core domain of D104G.

### Changes in collective motion

**Figure 5:**
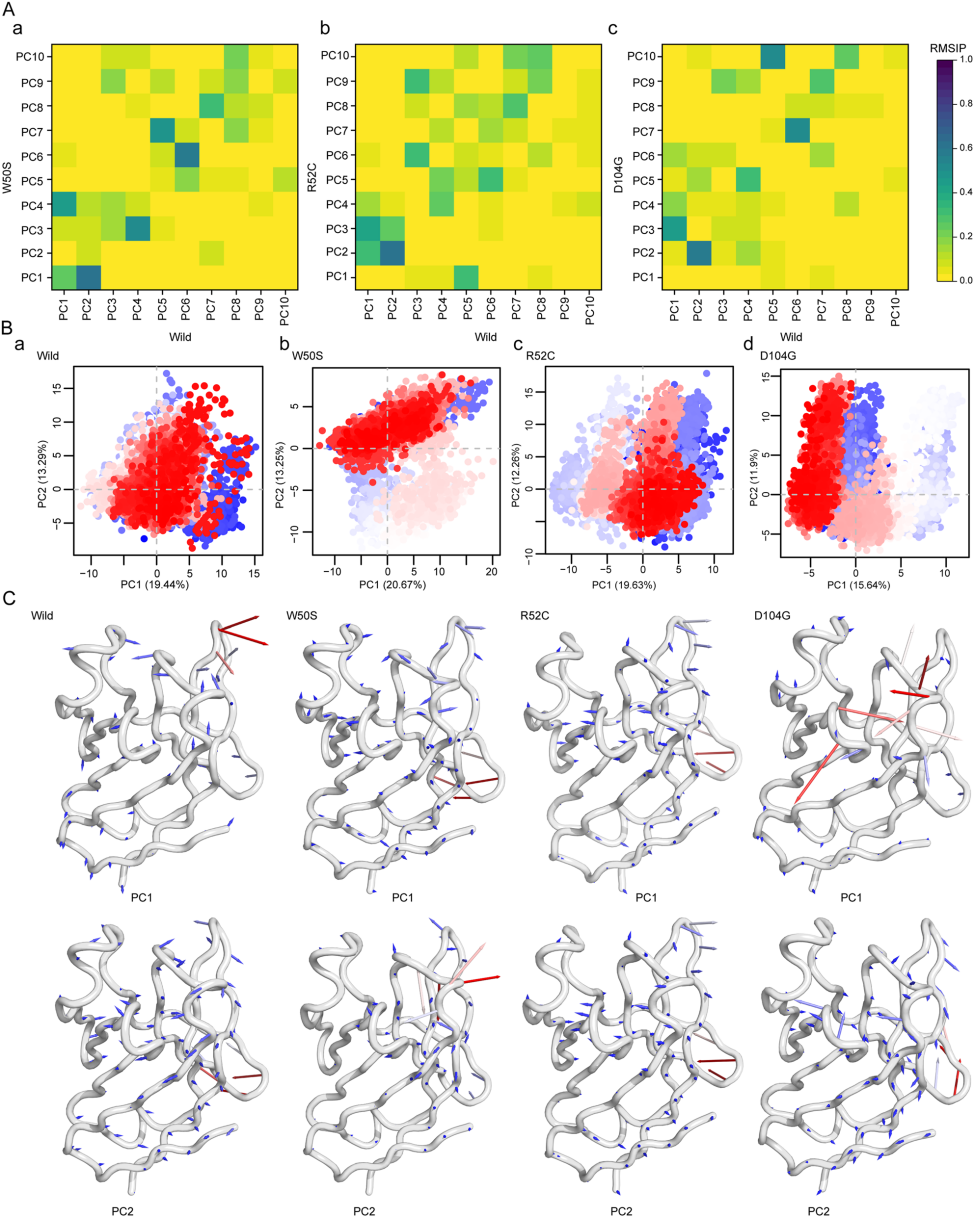
Changes protein dynamics induced by TREM2 variants were obtained by principal component analysis. (**A**) Root-Mean Square Inner Products (RMSIPs) of the first ten principal components showing similarities between the conformational spaces of the wild-type and variants. A gradient heat map indicating RMSIP values from yellow to dark blue. (**B**) The distribution of trajectory conformers was projected onto the principal planes of components 1 and 2, where dots represent the structure colored based on time of evolution (from blue to red). (**C**) Contributory movements in the first (upper panel) and second (lower panel) are represented by porcupine plots for all variants and wild-type.

Protein dynamics can be visualized by principal component analysis (PCA), which represents ensembles using a sequence of eigenvectors, whereby each eigenvector represents an aspect of protein motion by a phase space behavior^53^. PCA analysis (Fig. 5) showed the variances of variant structures differed from the wild-type. The similarities and differences between essential subspaces of wild-type and variant ensembles were highlighted by root mean square inner product (RMSIP) calculations^53–55^. Plots representing pairwise comparisons between the wild-type and variants suggested that the dynamic motions of the variants and wild-type differed, and substantial differences were observed for the first three principle components (PCs) (Fig. 5 Aa, b & c). In addition, PCA trace values of the wild-type, W50S, R52C, and D104G were 46.5, 70.492, 66.412, and 63.905 Å^2^, respectively, which indicated the variants had higher collective flexibilities than the wild-type^37^. The conformational distributions of protein structure in each subspace were projected onto a 2D plot for the first three PCs of wild-type and variant trajectories (Fig. 5B). The wild-type had more concentrated, overlapping dots on PC 1/2 projections than the variants (Fig. 5Ba and Fig. 5Bb, c&d), and projected directions of dot distributions also differed, suggesting the change of conformational behavior. Similar patterns were also observed in projections of PC 1/3 and 2/3, though differences were more subtle than those in wild-type and variant projections (Figure S3, Supplementary file 2). Color-coded scattered dots in PCA plots represent conformational states; red dots denote the steady-state, blue dots unstable states, and white dots intermediate states^56^. To visualize these changes in protein structure, a porcupine plot was generated for each variant and wild-type (Fig. 5B). Corresponding fluctuations are presented as line plots in Figure S4 (Supplementary file 2).

As shown in Figs. 5B and S3A (Supplementary file 2), dynamic changes in variant structures largely occurred in CDRs, as was shown by RMSF analysis. Furthermore, D104G caused a substantial fluctuation in the CDR1 region and in the βD-βE loop but reduced fluctuation in the CDR3 loop in PC1 (Figure S4A, Supplementary file 2). Notably, the wild-type structure conferred a stable transition in the CDR3 loop, and this was disrupted in the variants. W50S showed a large amplitude at the βC-βC' loop in PC1 but a lower amplitude in PC2 (Figure S4B, Supplementary file 2) and exhibited marked fluctuations in the CDR1 loop. R52C showed notable changes in the CDR2 and CDR3 regions of PC1 and PC2 as compared with the wild-type.

### Stability of secondary structures in the TREM2 Ig domain

**Figure 6:**
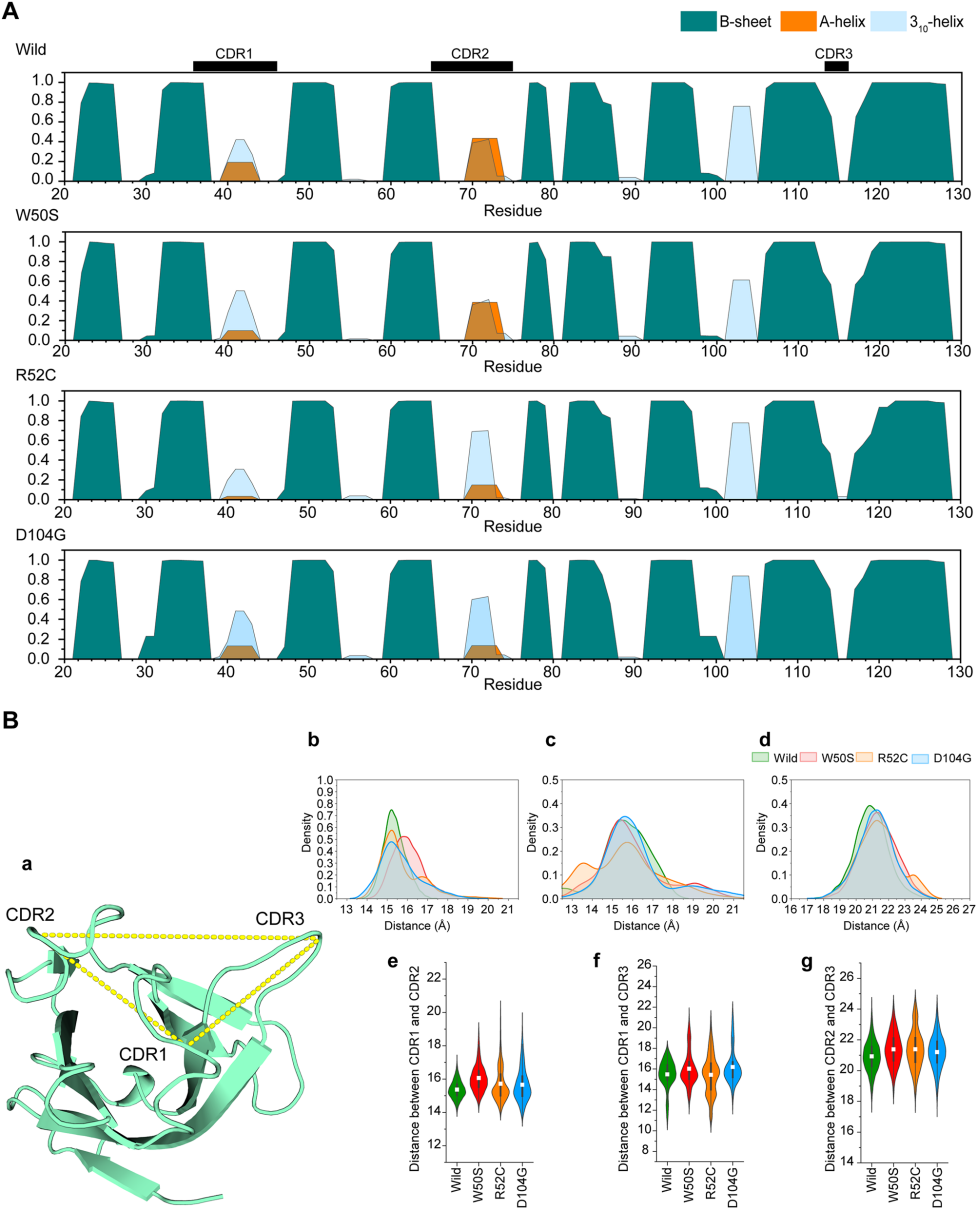
Structural alterations at CDRs due to the presence of nsSNPs. (**A**) Occupancies of secondary structures represented as fractions and percentages were calculated for α-helices (A-helix), β-sheets (B-sheet), and 3_10_-helices (3_10_-helix). (**B**) Changes in the inter-loop distances between CDRs. Distance calculations were performed on α-carbons of His43 (CDR1), Leu72 (CDR2), and Ser116 (CDR3) based on considerations of equilibrated trajectories (**a**). Changes in inter-loop distances represented as probability density functions for CDR1 and CDR2 (**b**), CDR1 and CDR3 (**c**), and CDR2 and CDR3 (**d**). Mean differences in distances between CDR1 and CDR2 (**e**), CDR1 and CDR3 (**f**), and CDR2 and CDR3 (**g**) are shown as violin plots.

Define Secondary Structure of Proteins (DSSP) is frequently used to identify changes in protein secondary structures during MD simulation. Since RMSF, DCCM, and PCA analyses (Figs. 3, 4 & 5) suggested variants induced significant alteration in CDR dynamics, we examined the total occupancy of essential secondary structures, including helix and strand formation, and contributions of residues in equilibrated trajectories. As shown in Fig. 6A, in the wild-type structure α-helix formation was observed in CDR1 and CDR2 loops with occupancies of ~ 20% and 40%, respectively. However, TREM2 variants exhibited less A-helix formation and more 3_10_-helix formation in CDRs and other regions, including residues 55–58, 87 to 91, and 101 to 106, respectively. For example, A-helix formation in W50S was 11% lower, but 3_10_-helix formation was 45%. In addition, a marked increase in 3_10_-helix occupancy was observed in the CDR2 region of R52C, while its C-terminal region (110 to 129) lost most of its β-strand structure. On the other hand, D104G exhibited increased β-strand structure formation, especially in 90 to 129.

### Stability of CDRs in the TREM2 Ig domain

Previous studies have reported that stability in CDR regions is critically maintained by intra-residual communication, which is typically altered by mutation^15,31,32^. As substantial alterations in the stability and structural organization of CDRs had been noted in the variants containing TREM2 structures, we monitored and plotted inter-loop distances among the α-carbons of CDR1, CDR2, and CDR3 (Fig. 6B). As shown by Fig. 6B, the distances between CDR1 and CDR2 (Fig. 6Bb) and between CDR1 and CDR3 (Fig. 6Bc) in all variants differed substantially from the wild-type. The distance between CDR2 and CDR3 in R52C showed a wide distribution, whereas in D104G showed a narrow distribution (Fig. 6Bd).

All variants exhibited a greater average distance between CDR1 and CDR2 and between CDR2 and CDR3 than the wild-type (Fig. 6Be&g). As regards, average distances between CDR1 and CDR3 in W50S and D104G were markedly greater than in R52C and the wild-type (Fig. 6Bf), which supported our fluctuation and motion analyses findings. An increase in average inter-loop distance suggests that increased motion caused CDRs to spread and disrupt electrostatics potentials near the PLIR, including CDR2 and the βC" strand.

Since the stability of CDR loops can be altered or achieved by inter-loop communication, total contact between CDR loops was counted and plotted in heatmap (Figure S5, Supplementary file 2). Total contact between any two residues of different loops was only considered when contact occupancy exceeded 10%. The results obtained showed inter-loop interaction between CDR1 and CDR2 (Figure S5A, Supplementary file 2) and CDR1 and CDR3 (FigureS4B, Supplementary file 2), and no interaction between CDR2 and CDR3. As can be seen in the wild-type, Arg47 of CDR1 maintained contact at ~ 100% with Thr66 to Asn68 residues of CDR2 (Figure S5Aa, Supplementary file 2), but different interaction patterns with Leu69 and Trp70 in variant containing structures. All three variants exhibited reduced contact formation between Asn68 and Gly45, but substantial inter-variant differences were observed for contacts between Trp70 and Lys42 or Arg47 (Figure S5A b, c & d, Supplementary file 2). As regards contact formation between the CDR1 and CDR3, the Arg46 residue of the CDR loop of R52C exhibited contact formation with Ser116 to Glu117, whereas the wild-type showed interactions only with His114 and Gly115. All variants exhibited substantially less contact between His114 and His43 or Lys42 than the wild-type, whereas R52C showed less contact with His43 (< 20% vs. > 40% for the wild-type).

### Changes in electrostatic potentials near the PLIR

**Figure 7:**
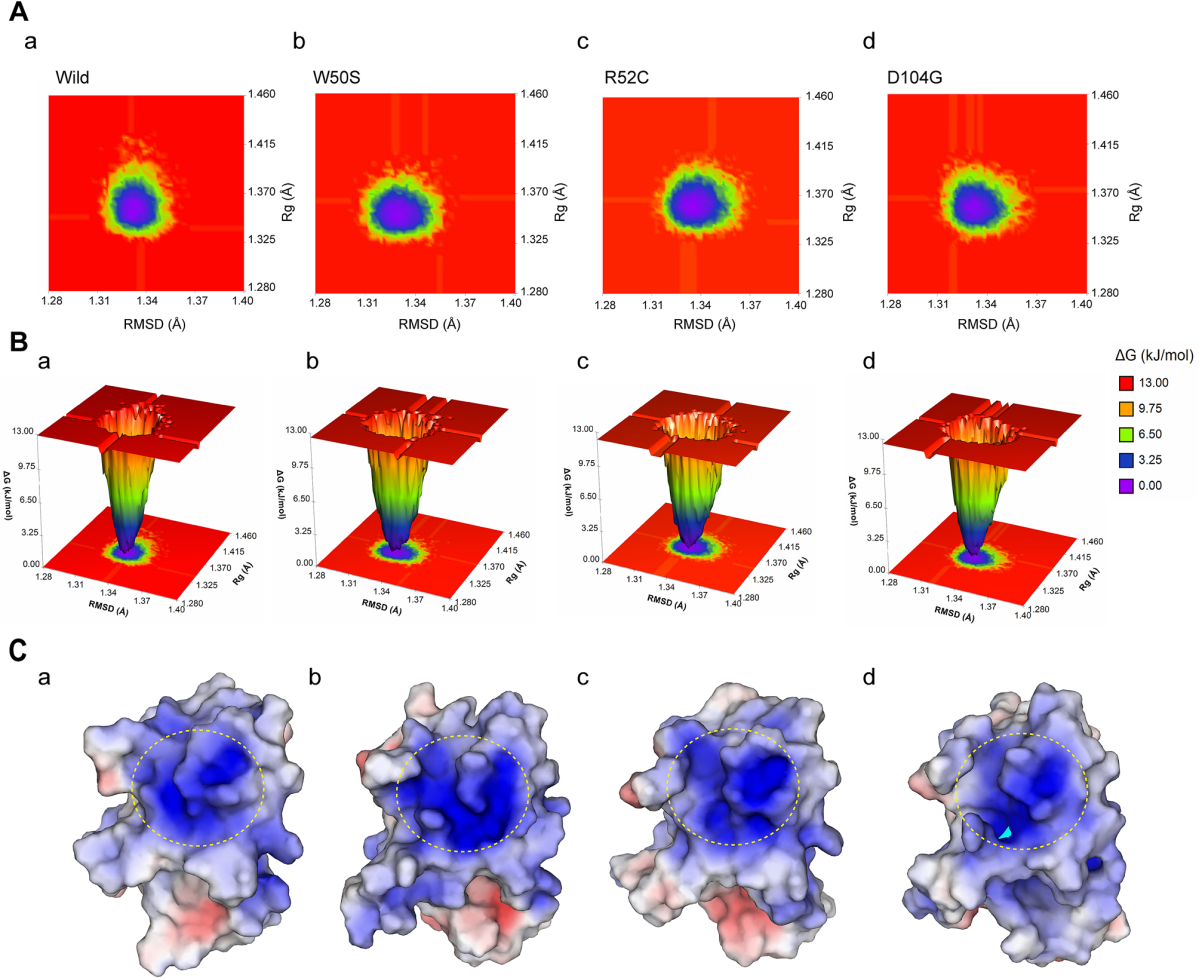
Effect of variants on the positive electrostatic potential near CDRs. (**A**) Free energy contour maps showing conformational distributions according to depth of energy minima for the wild-type (**a**), W50S (**b**), R52C (**c**), and D104G (**d**). Energy states as demonstrated by a color-coded map, where purple indicates a protein conformer with a lower energy minimum, and red a higher energy minimum then the wild-type. (**B**) Three-dimensional representation of the free energy landscapes (FEL) or the wild-type (**a**), W50S (**b**), R52C (**c**), and D104G (**d**). (**C**) Surface map of positive electrostatic potentials near the PLIR for the wild-type (**a**), W50S (**b**), R52C (**c**), and D104G (**d**) variants. The map was constructed using the lowest energy conformers retrieved from FEL, at corresponding timestamps of 292.5 (wild-type), 639.1 (W50S), 827.6 (R52C), and 868.1 ns (D104G). Darker blue regions indicate electropositive areas (> 6 kT/e), and red regions electronegative areas (< -6 kT/e). The yellow dotted line indicates the PLIR.

Electrostatic potentials over the solvated protein surface play an essential role in the recognition and binding of macromolecules. TREM2 disease-associated mutants have been reported to change electrostatic potentials near the PLIR^57^. We used free energy landscape analysis (FEL) to surface map electrostatic potentials near this region for all variants and wild-type structures. FEL represents conformational distributions from high to low energy minima^58,59^. In Fig. 7A, purple and red colors represent minima from low to high energy. Substantial changes in the conformational space region were observed in FEL maps of variants as compared with the wild-type (Fig. 7A). As shown in Fig. 7B, the wild-type structure showed narrow deeper purple grooves than the variant structures, whereas variant structures showed wider grooves, suggesting that variants exhibited conformational and structural transitions. Representative conformers with stable lowest energy states were extracted using FEL to draw electrostatic potential surface maps for each variant (Fig. 7C), and these maps showed subtle changes in wild-type electrostatic potentials (Figs. 7Ca, b, c &d). W50S (Fig. 7Cb) and R52C (Fig. 7Cc) variants increased electrostatic potential near the PLIR, which was absent in D104G (Fig. 7Cd). Furthermore, all variants had CDRs with helical conformations, indicating altered conformational stability in the ligand-binding region (Figure S6, Supplementary file 2).

## Discussion

Using a series of bioinformatic algorithms, the present study identified three potential deleterious SNPs, namely, W50S, R52C, and D104G, in TREM2 and confirmed their deleteriousness by dynamic behavior analysis. It is worth noting that the prediction of deleterious SNPs using a single tool can result in false positives, and thus, several algorithms, including sequence and structure-based approaches, were utilized to maximize prediction accuracy^43,44^. Accordingly, W50S, R52C, and D104G, were classified as high-risk SNPs, by consensus prediction. Interestingly, these three variants exhibited substantial changes in the CDR loops as compared TREM2. More specifically, they showed changes in dynamic motion, secondary structure, and increased inter-loop distances. Previous studies have reported a short α-helix in the CDR2 loop of AD-associated variants^15,31^ and suggested it might be associated with disease severity^32^. MD simulation showed that the CDR2 loop of the wild-type structure maintained an α-helix conformation in around 40% trajectories (Fig. 6A), which suggested the α-helix in the CDR2 loop is included the dynamic properties of the wild-type. However, in the TREM2 variants, the helical pattern was of the 3_10_-helix type (Fig. 6A), which indicates that this structural conversion might be associated with disease severity.

Several studies have provided evidence that disease-associated variants of TREM2 exhibit conformational remodeling of CDR^32^. This region comprises a hydrophobic patch mainly composed of residues 40–47 (CDR1), 67–78 (CDR2), and 115–120, which are essential for ligand binding specificity and strength. Furthermore, it has been suggested conformational remodeling depends on inter-loop communication and that nonbonded interactions between these loops modulate secondary conformation. CDR2 conformational stability is critically maintained by interactions between Ser65, Thr66, His67, and Asn68 and Arg47 (in the CDR1 loop)^31^. In the present study, these interactions differed in variants during simulation, except for interactions involving Leu71 (Figure S5, Supplementary file 2). Interestingly, distances between CDRs were greater in all variants, especially in W50S. It has been predicted, alterations of positive electrostatic potentials near the PLIR can increase CDR motion^15,60^, and thus, greater distances between CDRs might expose a buried patch of negative electrostatic potential and influence ligand binding. Analysis of a single protein structure obtained by FEL analysis revealed significant changes in secondary organizations and electrostatic potential surface area expansion (Fig. 7), indicating variants exposed residues typically buried in the wild-type structure. In the case of R52C, a marked change was observed, whereby increased flexibility induced significant correlated motion of CDRs (Fig. 4B), which increased nonbonded contacts between loops and facilitated conformational remodeling in CDR1 and CDR3 (Fig. 4B). Although these helix formations did not grossly change electrostatic potential surface areas (Fig. 7C), a substantial reduction in β-sheet formation was also observed near the CDR3 loop region (Fig. 6A). In a previous study, it was demonstrated that reductions in CDR stability reduced ligand binding, which it was argued would induce immune inactivation and possibly underlie the neurodegenerative effects of TREM2 variants^40^ (Table S4, Supplementary file 2).

Replacement of residues in proteins can change inter- and intra-molecular interactions and communications by altering protein flexibility and causing steric clashes and unfavorable interactions^38,61,62^. In the current study, we like others^31,32,37^, identified major changes in the inter-loop interactions of variants (Fig. 6B and Figure S5), which have been posited to underlie loss of CDR stability and function^39,40^. In the W50S, R52C, and D104G variants, substituted residues differed from those of the wild-type in hydrophobicity, charge, and size. Briefly, serine is smaller and more hydrophilic than tryptophan at 50, while cysteine in R52C is small, neutral in terms of charge, and more hydrophobic than the wild-type residue. Similarly, glycine is more hydrophobic, negatively charged, and more flexible than aspartic acid at 104. Increased hydrophilicity in W50S results in the loss of hydrophobic interactions with neighboring residues, whereas increased hydrophobicity in R52C and D104G disrupts ionic interactions and hydrogen bonding. Furthermore, W50S is buried in the core of the Ig domain, and the smaller size of serine might disturb core structure.

The findings of the present study agree well with previously published MD simulation findings and experimental data regarding loss of ligand binding in TREM2 variants due to the conformational remodeling of CDRs driven by alterations in inter-residue contacts, and thus, TREM2 binding site alterations (Table S4, Supplementary file 2). Actually, the in silico deleterious SNP prediction methods also identified several rare disease-associated TREM2 variants. The previously reported rare AD-associated missense variants R47H, H157Y and D87N (Supplementary file 1)^63^ were also identified as deleterious by more than five tools in the present study. R47H has deleterious characteristics similar to W50S, R52C, and D104G, including CDR conformational remodeling and loss of ligand binding in functional assays. In addition, the NHD and FTD associated missense variants Y38C, T66M, S31F, and R47C^64^ were identified as deleterious by most of the in silico tools used in this study, and Y38C and T66M were found experimentally to cause loss of ligand binding. In a molecular dynamics simulation study, it was suggested that Y38C and T66M cause severe conformational remodeling in CDRs loops by changing the interloop nonbonded interaction network and structural dynamics^15^. Interestingly, our MD simulation results for identified variants concurred with previously reported MD simulation results and experimental, which indicates that the methods used for physicochemical, structural, and dynamic characterizations reliably explained loss of ligand binding by TREM2 variants.

Although this in silico study provides detailed insight into the disruptive effects of nsSNPs, further biochemical and structural comparative studies on known variants are required to confirm our findings. In addition, long-timescale atomistic simulations and dynamic studies using replica exchange or other extensive sampling techniques are required to allow firm conclusions to be drawn as to whether nsSNPs functionally disrupt TREM2 in a background of neurodegeneration.

## Conclusion

The present study was conducted using a comprehensive bioinformatics design and identified three nsSNPs, namely, rs549402254 (W50S), rs749358844 (R52C), and rs1409131974 (D104G), in the *TREM2* from nsSNPs contained in the NCBI database, which induces structural alterations in the TREM2 Ig domain revealed by MD simulation. Detailed characterizations of the simulated trajectories of these variants demonstrated they exhibited increased loop motion and instability, particularly in CDRs. Although further experimental validation is required to confirm these variants cause reduced TREM2 ligand binding, the altered structural dynamics of TREM2 variants found in the present study concur with those reported for previously identified disease-associated TREM2 variants. We believe our findings reveal that in silico studies provide another means of revealing links between genetic-based studies and neurodegenerative disorders. Furthermore, the study provides new information on the dynamics of CDR regulations in wild-type TREM2 and its deleterious variants, and thus, provides clues regarding drug design and TREM2 gene therapy for the treatment of neurodegenerative diseases.

## Methods 

### Data collection and identification of deleterious SNPs of TREM2

Information regarding *TREM2* SNPs was retrieved using the NCBI SNP database with their corresponding rs IDs. As we focused exclusively on the identification of deleterious SNPs, 17 widely accepted in silico tools were used to identify high-risk SNPs in the *TREM2* gene. The prediction tools used were: SIFT, PolyPhen, Condel, FATHMM, DANN, CADD, M-CAP, Meta-LR, MutPred, MutationAssessor, PROVEAN, VEST3, fathmm-MKL, MuPRO, iStable, PhD-SNP, and SNAP2. Sorting Intolerant from Tolerant or SIFT is an analysis program based on the PSI-Blast algorithm^65^ that differentiates neutral and deleterious SNPs^66^ and predicts deleterious effects by applying the sequence homology approach^67^. Polyphen calculates the absolute value of differences between the profile scores of allelic variants in their polymorphic positions^68^. The number of aligned sequences is shown at the query position, and these aid evaluations of the reliabilities of profile score calculations^68^. Condel is an in silico tool used to validate the outcomes of nonsynonymous single nucleotide variants SNVs based on the ensemble scores of multiple prediction tools (SIFT, Polyphen2, MutationAssessor, and FATHMM)^69^. The prediction result is denoted by a score between 0 to 1, which are considered to be deleterious or neutral, respectively^70^. CADD provides a comprehensive evaluation of variants using C-scores or "Phred" scores^71^. CADD score is described as a "meta-annotation" tool because it utilizes data from many other functional annotation tools^72^ and can effectively predict a variant's effect on a protein^73^. DANN utilizes a DNN (deep neural network) algorithm that captures non-linear relationships among Boolean features defined for each variant^74^. This allows DANN to annotate non-coding variants and prioritize putative causal variants^74^. FATHMM provides an efficient species-independent method and utilizes large-scale genome sequencing projects with functional, molecular, and phenotypic associations^75^.fathmm-MKL is an integrated algorithm based on the Hidden Markov model and predicts the functional effects of non-coding and coding sequence variants^76^. The pathogenicity classifier tool M-CAP exhibits 95% sensitivity at dismissing 60% of rare missense variants of uncertain significance^77^. MutPred can predict the pathogenicity of amino acid substitution and the disease mechanism by employing Random forest depending on the amino acid sequence, functional properties, calculated protein structure and dynamics, and evolutionary information^78^. The prediction is expressed as a probability of deleterious effect, and a probability of > 50% denotes pathogenicity^79^. MutationAssessor predicts based on the evolutionary conservancy of affected residues and predicts the possible role of a mutation on phenotype using multiple sequence alignment^80,81^. Functional impact scores are generated from this evaluation of evolutionary information (FIS) and categorize nsSNP as neutral, low, medium, or high^81^. PROVEAN^82^ is a high throughput online prediction tool that utilizes an alignment-based approach for single and multiple amino acid insertions, deletions, and substitutions to identify disease causing variants^83^. VEST3 uses a machine-learning classifier, which ranks rare missense variants by probability of disease association^84^. VEST3 is based on a random forest algorithm that identifies functional missense mutations^85^ and provides p-values for false discovery rates (FDR)^86^. MuPro (a web tool) was used to predict nsSNP-induced alterations in protein stability from energy change values^87^, which are quantified using confidence scores that range between − 1 and 1, where a score of < 0 indicates a decrease in protein stability, and a score of > 0 indicates an increase in protein stability^88,89^. The degree of protein destabilization and free energy variation was calculated using iStable^90^ (http://predictor.nchu.edu.tw/istable/indexSeq.php) and a support vector machine (SVM). iStable predicts changes in protein stability caused by a single amino acid residue mutation^91^. PhD-SNP^92^ is another tool that utilizes SVM to predict neutral or disease-associated SNPs^93^. Based on a neural network classifier, SNAP2 predicts nsSNP-induced changes in secondary structure^94^, and distinguishes between the effects of neutral to deleterious SNPs by considering the solvent accessibility effect, secondary structure, and evolutionary conservation^95^.

### Molecular dynamics (MD) simulation

The crystal structure of the TREM2 Ig domain was downloaded from the RCSB protein databank (PDB ID: 5UD7) and then prepared in Schrödinger 2017–1 (Schrödinger, LLC, New York, NY, USA, 2017), as previously described^15,59,96–99^. After preparing the structure, Schrödinger 2017–1 (Schrödinger, LLC, New York, NY, USA, 2017) was used to include the three variants (W50S, R52C, and D104G) in the structure using mutant residue script. This was followed by a short MD simulation to refine and minimize structure energies using YAMBER3 force field^100,101^ in YASARA Dynamics software (YASARA Biosciences GmBH, Vienna, Austria), as previously described^44,52,96^. Further analyses were then conducted using the lowest energy MD conformer.

MD simulation was conducted using YASARA (YASARA Biosciences GmBH, Vienna, Austria) dynamics software, hydrogen bond network optimization^96,98,99,102,103^, and AMBER14 to apply force fields^100,101^. A simulation cell (Cubic box) was prepared in a periodic boundary condition and extended on each side such that it was 10 Å larger than the selected protein. The water model TIP3P (transferable intermolecular potential3 points) was added to solvate the whole system using a solvent density of 0.997 gL^−1^^104^. pKa (acid dissociation constant) values of protein residues were evaluated in the solvated state. Neutralization was performed by adding Na^+^ or Cl^−^ ions to the cubic cell to maintain a physiological concentration of 0.9% (0.15 M NaCl). The SCWRL algorithm and H-bonding network optimization were used to carry on the protonation state accurately for each specific amino acid^105^. A simulated a nnealing method was followed by minimizing the energy of each simulated system using the steepest gradient approach for 5000 cycles. Time step interval was fixed at 2.00 fs for all simulations using a multiple time-step (MTS) algorithm. MD simulation was then run at a NaCl concentration of 0.9%, pH 7.4, and 298 K using PME (particle-mesh Ewald), and an 8 Å cut-off distance was used to calculate long-range electrostatic interactions while maintaining the periodic boundary condition^106^. The Berendsen thermostat was applied at constant pressure to maintain the temperature of the simulation system, and time was incremented at 2.00 fs^107–109^. Three independent 500 ns MD runs were performed for each system, and results were obtained using a time interval of 100 ps for all simulated trajectories. Results were analyzed using RMSF and RMSD. We also analyzed SASAs of protein backbones and Rg values using VMD (Version 1.9.3) software^110,111^ developed from the default script of YASARA^109^.

### Dynamic cross-correlation maps (DCCMs)

DCCMs were calculated to explore the linked motions of all Cα atoms in equilibrated trajectories for all variants and wild-type. The Bio3D^112^ package integrated into the R program was used to analyze DCCMs and provided Pearson's covariance matrix correlation coefficients, known as "cov2dccm" coefficients. C_ij_ (the cross-correlation ratio) was determined for Cα electrons^113^ using the following equation,

$${{C}}_{\mathrm{ij}}=\frac{\langle \Delta {\mathrm{r}}_{\mathrm{i}}\,\,.\,\, \Delta {\mathrm{r}}_{\mathrm{j}}\rangle }{({{\langle \Delta {\mathrm{r}}_{\mathrm{i}}^{2}\rangle }{\langle \Delta {\mathrm{r}}_{\mathrm{j}}^{2}\rangle }})^{1/2}}$$

where ∆r_i_ and ∆r_j_ are average location s of the ith and jth atoms, respectively. C_ij_ values range from − 1 to + 1, where positive values indicate degrees of correlated motion between residues *i* and *j*, and negative values indicate degrees of anticorrelated motion.

### Principal component analysis (PCA)

To understand atomic movements and protein loop dynamics, PCA was implemented by calculating and diagonalization of the atomic coordinates of eigenvectors and positional covariance matrices based on eigenvectors and equal eigenvalues, which amplify the displacements of atoms in MD simulation trajectories^114,115^. The Bio3D package^112^ was used for PCA as we previously described^96^.

In addition, root mean square inner products (RMSIPs) were calculated using the first three principal components to obtain similarities between two sets of modes from normal modes or principal components. Here, the range of RMSIP values was set from 0 to 1, where 0 indicates orthogonal directionality and 1 indicates identical directionalities of sample subspaces^47,116^; values were calculated using Bio3D^54^. Also, the essential dynamics program of GROMACS was used to calculate the cosine content of principal components obtained from each simulation^117^.

### Free energy landscape (FEL)

Gibb's free energies, a mapping system, and free energy landscape (FEL) analysis were used to identify most stable states from protein conformations; FEL explains energy distributions of protein folding throughout MD simulations^118,119^. FEL was also used to obtain protein enthalpy and entropy functions^97^. The following equation was used to determine Gibb's free energy landscapes,

$${\text{G}}_{{\text{i}}} \, = -{\text{K}}_{{\text{B}}} {\text{Tln }}({\text{N}}_{{\text{i}}} /{\text{N}}_{{{\text{max}}}} )$$

Here, K_B_ is Boltzmann's constant, and the temperature (T) was set at 300 K. N_i_ represents the population of bin I, and N_max_ represents the most occupied bin. Different color codes were generated to depict maximum and minimum energy levels. Based on radius of gyration and RMSD values, free energy contour maps were constructed for the most stable energy conformers.

## Supplementary Information

Supplementary Video 1.

Supplementary Video 2.

Supplementary Video 3.

Supplementary Video 4.

Supplementary Information 1.

Supplementary Information 2.
