# Supplementary material for: Dynamic insights into the effects of nonsynonymous polymorphisms (nsSNPs) on loss of TREM2 function
Source: Sci Rep. 2022 Jun 7;12:9378. doi: 10.1038/s41598-022-13120-5 (PMC9174165; doi:10.1038/s41598-022-13120-5)
Supplement: Supplementary file 5 — Supplementary Information 1. [file 41598_2022_13120_MOESM5_ESM.docx]

**Supplementary File 1**

**Dynamic insights into the effects of nonsynonymous polymorphisms (nsSNPs) on loss of TREM2 function**

Raju Dash^1^, Yeasmin Akter Munni^1^, Sarmistha Mitra^1^, Ho Jin Choi^1^, Sultana Israt Jahan^2^, Apusi Chowdhury^3^, Tae Jung Jang^4^, Il Soo Moon^1^

^1^Department of Anatomy, Dongguk University College of Medicine, Gyeongju, 38066, Republic of Korea

^2^Department of Biotechnology and Genetic Engineering, Noakhali Science and Technology University, Noakhali, 3814, Bangladesh.

^3^Department of Pharmaceutical Science, North-South University, Dhaka-12 29, Bangladesh

^4^Department of Pathology, Dongguk University College of Medicine, Gyeongju 38066, Republic of Korea

* Correspondence:

Il Soo Moon

Email: [moonis@dongguk.ac.kr](mailto:moonis@dongguk.ac.kr)

List of nsSNPs of *TREM2* and their deleterious prediction by *in silico* tools

| **rs ID** | **Protein position** | **Amino acids** | **SIFT** | **PolyPhen** | **Condel** | **CADD** | **DANN** | **FATHMM** | **M-CAP** | **MetaLR** | **MutPred** | **MutationAssessor** | **PROVEAN** | **VEST3** | **fathmm-MKL** | **MuPro** | **iStable** | **PhD-SNP** | **SNAP2** |
| --- | --- | --- | --- | --- | --- | --- | --- | --- | --- | --- | --- | --- | --- | --- | --- | --- | --- | --- | --- |
| rs753777378 | 204 | T/A | 0.33 | 0 | 0.024 | 0.074 | 0.337045 | 1.14 | 0.001739 | 0.0322 | 0.283 | - | - | 0.007 | 0.01111 |  | Decrease |  |  |
| rs200820365 | 183 | S/C | 0.04 | 0.54 | 0.532 | 7.301 | 0.841593 | 0.96 | 0.003088 | 0.0425 | - | - | - | 0.065 | 0.02527 |  | Decrease |  |  |
| rs75272959 | 177 | E/K | 0.1 | 0.058 | 0.29 | 2.11 | 0.592472 | 1 | - | 0.0077 | - | - | - | 0.065 | 0.01504 |  | Decrease |  |  |
| rs199795809 | 166 | V/M | 0.27 | 0.014 | 0.034 | 11.75 | 0.866913 | 0.93 | 0.004102 | 0.0229 | - | - | -0.23 | - | 0.00156 |  | Decrease |  |  |
| rs138355759 | 223 | T/I | 0.34 | 0.003 | 0.023 | 0.032 | 0.685994 | 1.11 | 0.002612 | 0.0288 | - | 1.405 | -2.17 | 0.085 | 0.04478 |  | Increase |  |  |
| rs150277350 | 192 | A/T | 0.13 | 0.085 | 0.259 | 9.421 | 0.813135 | 1.07 | 0.006973 | 0.0405 | - | - | -0.29 | 0.146 | 0.00918 | -0.7293 | Decrease | 0.454 | -46 |
| rs150277350 | 205 | R/H | 0.63 | 0 | 0.004 | 9.421 | 0.813135 | 1.07 | 0.006973 | 0.0405 | - | - | -0.29 | 0.146 | 0.00918 |  | Decrease |  |  |
| rs28937876 | 186 | K/N | 0 | 0.946 | 0.835 | 26.2 | 0.998275 | 1.2 | 0.026249 | - | - | - | - | 0.265 | 0.59734 | -0.56252 | Decrease | 0.658 | 91 |
| rs28937876 | 200 | D/Y | 0.04 | 0.065 | 0.353 | 26.2 | 0.998275 | 1.2 | 0.026249 | - | - | - | - | 0.265 | 0.59734 |  | Decrease |  |  |
| rs781302866 | 172 | P/S | 0.13 | 0.086 | 0.259 | 13.82 | 0.971778 | 0.96 | 0.005338 | 0.0342 | 0.464 | - | -1.62 | 0.095 | 0.12359 | -0.0251 | Decrease | 0.322 | -26 |
| rs781302866 | 185 | T/I | 0.01 | 0.027 | 0.407 | 13.82 | 0.971778 | 0.96 | 0.005338 | 0.0342 | 0.464 | - | -1.62 | 0.095 | 0.12359 |  | Decrease |  |  |
| rs138788407 | 170 | F/L | 0.27 | 0 | 0.034 | 0.867 | 0.737186 | 1.39 | 0.004162 | 0.0151 | - | - | -0.3 | 0.233 | 0.15022 | -0.74212 | Decrease | 0.246 | -51 |
| rs138788407 | 184 | P/A | 0.19 | 0.003 | 0.05 | 0.867 | 0.737186 | 1.39 | 0.004162 | 0.0151 | - | - | -0.3 | 0.233 | 0.15022 |  | Decrease |  |  |
| rs267601028 | 166 | G/R | 0.37 | 0.01 | 0.019 | 9.188 | 0.595407 | 1.13 | 0.004645 | 0.0193 | 0.364 | - | -0.91 | 0.257 | 0.01283 | 0.188767 | Decrease | 0.223 | -16 |
| rs267601028 | 179 | R/K | 0.51 | 0.377 | 0.13 | 9.188 | 0.595407 | 1.13 | 0.004645 | 0.0193 | 0.364 | - | -0.91 | 0.257 | 0.01283 |  | Decrease |  |  |
| rs2234255 | 157 | H/Y | 0.01 | 0.67 | 0.643 | 23.1 | 0.996327 | 1.11 | - | 0.1059 | - | 1.845 | -1.33 | 0.133 | 0.71531 | 0.673464 | Increase | 0.39 | 76 |
| rs79011726 | 151 | E/K | 0.03 | 0.502 | 0.532 | 23.2 | 0.998195 | 1.02 | 0.012985 | 0.077 | - | 2.255 | -1.23 | 0.5 | 0.09707 | -1 | Increase | 0.478 | 22 |
| rs149622783 | 136 | R/P | 0.1 | 0.318 | 0.386 | 12.03 | 0.894474 | 1.19 | 0.009238 | 0.0182 | 0.448 | - | 0.21 | 0.154 | 0.10949 | -0.93215 | Decrease | 0.659 | -13 |
| rs149622783 | 136 | R/Q | 1 | 0.001 | 0 | 1.841 | 0.505073 | 1.51 | 0.003662 | 0.0056 | - | -1.955 | 1.05 | 0.068 | 0.00056 | -1 | Decrease | 0.073 | -49 |
| rs28939079 | 134 | D/G | 0.03 | 0.736 | 0.633 | 7.798 | 0.99094 | 1.14 | 0.008356 | 0.0812 | 0.808 | 1.78 | -0.43 | 0.572 | 0.08202 | -1 | Decrease | 0.766 | 56 |
| rs139607688 | 131 | D/E | 0.16 | 0.294 | 0.15 | 12.45 | 0.986425 | 1.08 | 0.005265 | 0.0494 | 0.555 | 1.95 | -0.93 | 0.03 | 0.84439 | 0.087178 | Increase | 0.505 | -37 |
| rs201280312 | 130 | A/V | 0.14 | 0.242 | 0.274 | 10.98 | 0.978897 | 1.25 | 0.006104 | 0.0396 | - | 1.085 | -0.35 | 0.112 | 0.03167 | 0.250999 | Increase | 0.438 | -62 |
| rs121908402 | 126 | V/G | 0 | 0.998 | 0.919 | 28.2 | 0.997383 | 0.81 | 0.089734 | 0.2092 | 0.698 | 2.9 | -4.92 | 0.981 | 0.92553 | -1 | Decrease | 0.758 | 68 |
| rs145080901 | 105 | A/V | 0 | 0.997 | 0.911 | 24.2 | 0.998519 | -0.21 | 0.117236 | 0.3498 | - | 2.395 | -3.27 | 0.584 | 0.19067 | 0.266103 | Increase | 0.381 | 28 |
| rs147564421 | 98 | R/W | 0 | 0.897 | 0.798 | 25.2 | 0.998555 | -0.24 | 0.074285 | 0.2104 | - | 2.255 | -4.25 | 0.474 | 0.05298 | -0.15583 | Decrease | 0.522 | 40 |
| rs2234253 | 96 | T/M | 0 | 0.999 | 0.935 | 24.3 | 0.998123 | -0.42 | 0.463496 | 0.4809 | 0.365 | 3.465 | -4.06 | 0.853 | 0.8522 | 0.40444 | Increase | 0.583 | 52 |
| rs2234253 | 96 | T/R | 0 | 0.999 | 0.935 | 24 | 0.986359 | -0.31 | 0.328875 | 0.4235 | 0.17 | 2.57 | -4.41 | 0.723 | 0.87856 | -0.201 | Decrease | 0.702 | 56 |
| rs142232675 | 87 | D/N | 0.28 | 0.998 | 0.506 | 22.8 | 0.996315 | 1.63 | 0.397644 | 0.0566 | - | 1.425 | -2.18 | 0.119 | 0.29148 | -0.56559 | Increase | 0.355 | -6 |
| rs368255898 | 85 | T/I | 0.34 | 0.125 | 0.034 | 4.097 | 0.989073 | 1.92 | 0.006882 | 0.0166 | 0.241 | 1.59 | -1.31 | 0.267 | 0.02218 | -0.48102 | Decrease | 0.43 | 7 |
| rs368255898 | 85 | T/K | 0.29 | 0.415 | 0.164 | 14.5 | 0.965727 | 1.91 | 0.013024 | 0.0164 | - | 1.165 | -0.59 | 0.333 | 0.03135 | -0.84422 | Decrease | 0.584 | -22 |
| rs753372932 | 68 | N/K | 0.66 | 0.137 | 0.014 | 8.979 | 0.963001 | -0.13 | 0.011365 | 0.0433 | 0.405 | 0.75 | -0.09 | 0.055 | 0.05632 | -0.02733 | Decrease | 0.385 | -14 |
| rs201258663 | 66 | T/M | 0 | 0.999 | 0.935 | 29.4 | 0.999245 | -0.22 | 0.404729 | 0.4676 | - | 3.275 | -3.48 | 0.766 | 0.90361 | -0.29092 | Decrease | 0.544 | 44 |
| rs143332484 | 62 | R/L | 0.46 | 0.668 | 0.248 | 12.7 | 0.985973 | 0.1 | 0.029566 | 0.0713 | 0.635 | 1.02 | -1.51 | 0.487 | 0.02995 | -0.69988 | Decrease | 0.618 | -6 |
| rs143332484 | 62 | R/H | 0.37 | 0.049 | 0.022 | 11.11 | 0.966301 | -0.11 | - | 0.0486 | - | 0.61 | -0.13 | 0.05 | 0.01086 | 1 | Decrease | 0.216 | -22 |
| rs201258314 | 62 | R/C | 0.06 | 0.917 | 0.691 | 25.5 | 0.999387 | -0.12 | 0.069083 | 0.3035 | 0.663 | 2.34 | -3.08 | 0.416 | 0.90354 | -0.80516 | Decrease | 0.61 | 16 |
| rs886042808 | 58 | G/A | 0.1 | 0.975 | 0.704 | 19.44 | 0.993273 | -0.24 | 0.048073 | 0.229 | 0.652 | 1.895 | -1.88 | 0.342 | 0.05742 | -0.49729 | Decrease | 0.464 | 27 |
| rs886042808 | 58 | G/D | 0.06 | 0.569 | 0.518 | 14.25 | 0.996661 | -0.25 | 0.040503 | 0.1627 | 0.638 | 1.89 | -2.38 | 0.357 | 0.04113 | -0.85082 | Decrease | 0.663 | 58 |
| rs200392967 | 39 | D/E | 0.08 | 0.648 | 0.527 | 23.4 | 0.997825 | 2 | 0.006921 | 0.017 | - | 0.855 | -1.41 | 0.236 | 0.86359 | 0.163985 | Increase | 0.333 | -31 |
| rs764816591 | 39 | D/N | 0.7 | 0.041 | 0.005 | 12.85 | 0.951216 | 1.99 | 0.008489 | 0.0075 | 0.435 | 0.225 | -0.52 | 0.046 | 0.03487 | 0.019283 | Increase | 0.101 | -80 |
| rs797044603 | 38 | Y/C | 0 | 0.999 | 0.935 | 25.3 | 0.998387 | -0.34 | 0.289336 | 0.4837 | 0.727 | 3.66 | -8.69 | 0.972 | 0.84632 |  | Decrease |  |  |
| rs2234252 | 28 | A/V | 0.26 | 0.031 | 0.036 | 0.001 | 0.679492 | -0.08 | 0.020347 | 0.0264 | - | 0.195 | -0.74 | 0.088 | 0.01658 | 1 | Decrease | 0.306 | -68 |
| rs146485698 | 5 | R/Q | 0.65 | 0.492 | 0.167 | 12.61 | 0.99786 | 1.35 | 0.011817 | 0.0197 | - | 1.79 | -0.39 | 0.083 | 0.04013 | -0.48445 | Decrease | 0.052 | 1 |
| rs199759237 | 3 | P/L | 0.04 | 0.898 | 0.704 | 13.54 | 0.995651 | 1.1 | 0.012028 | 0.0832 | - | 2.215 | -3.32 | 0.116 | 0.03668 | -0.06748 | Decrease | 0.205 | -21 |
| rs141301187 | 3 | P/T | 0.03 | 0.301 | 0.458 | 0.212 | 0.965389 | 1.15 | 0.011155 | 0.0393 | - | 1.12 | -3.16 | 0.127 | 0.08467 | -1 | Decrease | 0.06 | -87 |
| rs560988938 | 213 | T/I | 0.09 | 0 | 0.296 | 0.697 | 0.595288 | 1.07 | 0.003663 | 0.0268 | 0.368 | - | - | 0.149 | 0.01126 |  | Increase |  |  |
| rs754547724 | 203 | T/A | 0.98 | 0 | 0 | 1.746 | 0.739148 | 1.14 | 0.002049 | 0.0295 | 0.286 | - | - | 0.022 | 0.00053 |  | Decrease |  |  |
| rs530314472 | 202 | E/D | 0.34 | 0.037 | 0.025 | 5.984 | 0.96116 | 1.03 | 0.005122 | 0.0344 | - | - | - | 0.057 | 0.02034 |  | Decrease |  |  |
| rs752403953 | 202 | E/Q | 0.21 | 0.075 | 0.049 | 2.956 | 0.855838 | 0.97 | 0.005327 | 0.0342 | 0.277 | - | - | 0.028 | 0.04573 |  | Decrease |  |  |
| rs746283732 | 198 | L/P | 0 | 0.027 | 0.446 | 5.569 | 0.826694 | 0.8 | 0.007271 | 0.0306 | 0.469 | - | - | 0.278 | 0.45776 |  | Decrease |  |  |
| rs749732493 | 191 | W/C | 0.06 | 0.005 | 0.323 | 3.477 | 0.501861 | 0.88 | 0.007215 | 0.0285 | 0.53 | - | - | 0.08 | 0.03466 |  | Decrease |  |  |
| rs532556414 | 190 | T/I | 0.48 | 0 | 0.011 | 4.352 | 0.862985 | 1.07 | 0.001912 | 0.0201 | 0.402 | - | - | 0.013 | 0.03061 |  | Increase |  |  |
| rs748257648 | 186 | C/Y | 0.29 | 0.022 | 0.03 | 3.33 | 0.563132 | 0.93 | 0.002107 | 0.0331 | 0.297 | - | - | 0.098 | 0.04534 |  | Increase |  |  |
| rs377430936 | 184 | P/Q | 0.01 | 0.839 | 0.726 | 4.287 | 0.972897 | 0.85 | 0.003827 | 0.1282 | 0.43 | - | - | 0.088 | 0.05301 |  | Decrease |  |  |
| rs376505321 | 181 | G/A | 0.02 | 0.271 | 0.412 | 1.897 | 0.511111 | 0.81 | 0.002007 | 0.0102 | - | - | - | 0.072 | 0.06127 |  | 0.5 |  |  |
| rs752213350 | 180 | P/S | 0.04 | 0 | 0.349 | 1.154 | 0.575526 | 0.9 | 0.006081 | 0.0094 | 0.207 | - | - | 0.027 | 0.02375 |  | Decrease |  |  |
| rs752213350 | 180 | P/A | 0.56 | 0 | 0.006 | 0.22 | 0.589895 | 0.92 | 0.005748 | 0.0259 | 0.226 | - | - | 0.028 | 0.03691 |  | Increase |  |  |
| rs534753905 | 172 | R/W | 0.09 | 0.889 | 0.646 | 4.271 | 0.983374 | 0.89 | 0.00713 | 0.0942 | 0.183 | - | - | 0.199 | 0.06886 |  | Decrease |  |  |
| rs368921728 | 230 | T/M | 0 | 0.003 | 0.445 | 17.36 | 0.957149 | 0.92 | 0.004596 | 0.0338 | - | - | -1.01 | 0.134 | 0.019 | -0.08989 | Decrease | 0.048 | 21 |
| rs755504623 | 162 | A/G | 0.12 | 0.015 | 0.261 | 0.077 | 0.960346 | 1.08 | 0.008133 | 0.038 | 0.236 | - | -0.62 | 0.045 | 0.06723 |  | Decrease |  |  |
| rs755504623 | 227 | L/V | 0.22 | 0.034 | 0.044 | 0.077 | 0.960346 | 1.08 | 0.008133 | 0.038 | 0.236 | - | -0.62 | 0.045 | 0.06723 | -0.80684 | Decrease | 0.088 | 11 |
| rs748227970 | 226 | G/E | 0.3 | 0.009 | 0.029 | 14.23 | 0.943806 | 0.64 | - | 0.0223 | 0.098 | - | -1.03 | 0.125 | 0.04454 | 1 | Increase | 0.18 | 23 |
| rs747067917 | 218 | G/R | 0.01 | 0.871 | 0.744 | 24.8 | 0.998342 | 0.95 | 0.003876 | 0.1113 | 0.712 | 2.085 | -1.1 | 0.588 | 0.04372 | -0.15301 | Decrease | 0.692 | 5 |
| rs373137055 | 216 | D/Y | 0.01 | 0.088 | 0.412 | 17.75 | 0.925508 | 0.82 | 0.00626 | 0.0455 | - | 1.905 | -2.64 | 0.359 | 0.01964 | 0.249568 | Increase | 0.554 | 29 |
| rs745696322 | 206 | H/Q | 0.34 | 0.013 | 0.024 | 0.044 | 0.773927 | 1.2 | 0.004258 | 0.0182 | 0.221 | - | 0.5 | 0.077 | 0.02362 | -0.11612 | Decrease | 0.178 | -12 |
| rs745696322 | 220 | S/T | 0.52 | 0.107 | 0.017 | 0.044 | 0.773927 | 1.2 | 0.004258 | 0.0182 | 0.221 | - | 0.5 | 0.077 | 0.02362 |  | Decrease |  |  |
| rs750046115 | 205 | R/C | 0.02 | 0 | 0.383 | 9.873 | 0.624335 | 1.18 | 0.002525 | - | - | - | - | - | 0.00548 |  | Decrease |  |  |
| rs541549712 | 190 | A/D | 0 | 0.655 | 0.675 | 23.7 | 0.993505 | 0.88 | 0.022576 | 0.0782 | 0.623 | - | -2.48 | 0.761 | 0.06479 | -0.46302 | Decrease | 0.881 | 30 |
| rs541549712 | 203 | S/R | 0.39 | 0.022 | 0.017 | 23.7 | 0.993505 | 0.88 | 0.022576 | 0.0782 | 0.623 | - | -2.48 | 0.761 | 0.06479 |  | Increase |  |  |
| rs560925779 | 190 | A/T | 0.02 | 0.299 | 0.476 | 15.48 | 0.9739 | 0.92 | 0.011013 | 0.053 | 0.527 | - | -0.94 | 0.41 | 0.03551 | -0.868 | Decrease | 0.67 | -46 |
| rs560925779 | 203 | S/N | 0.07 | 0.571 | 0.514 | 15.48 | 0.9739 | 0.92 | 0.011013 | 0.053 | 0.527 | - | -0.94 | 0.41 | 0.03551 |  | Decrease |  |  |
| rs751726815 | 180 | A/D | 0 | 0.881 | 0.788 | 27.6 | 0.997226 | 0.48 | 0.018129 | 0.1509 | 0.589 | - | -3.24 | 0.891 | 0.72321 | 0.032809 | Increase | 0.751 | 44 |
| rs371702633 | 162 | S/R | 0.08 | 0.079 | 0.305 | 0.591 | 0.853432 | 1.24 | 0.006509 | - | - | - | - | 0.036 | 0.0421 | 0.881353 | Increase | 0.326 | -15 |
| rs371702633 | 176 | P/A | 0.03 | 0.025 | 0.365 | 0.591 | 0.853432 | 1.24 | 0.006509 | - | - | - | - | 0.036 | 0.0421 |  | Decrease |  |  |
| rs539332069 | 161 | R/W | 0 | 0.995 | 0.902 | 29.7 | 0.999032 | 1.19 | 0.029706 | 0.2011 | 0.516 | 2.295 | -1.3 | 0.497 | 0.90351 | 0.399644 | Increase | 0.619 | 64 |
| rs750556369 | 149 | S/G | 0.03 | 0.011 | 0.365 | 0.136 | 0.974648 | 1.08 | 0.003756 | 0.0327 | 0.429 | 2.175 | -1.79 | 0.096 | 0.02124 | -0.65698 | Decrease | 0.179 | -7 |
| rs752812458 | 128 | V/L | 0 | 0.99 | 0.886 | 32 | 0.998116 | 0.79 | 0.038811 | 0.272 | 0.62 | 3.07 | -1.86 | 0.771 | 0.90761 | -0.07599 | Decrease | 0.644 | 31 |
| rs751357650 | 115 | G/D | 0.15 | 1 | 0.741 | 22.8 | 0.996875 | -0.19 | 0.018635 | 0.1484 | 0.596 | 1.09 | -1.48 | 0.441 | 0.04754 | 0.692791 | Increase | 0.553 | -1 |
| rs750002795 | 113 | L/V | 0.14 | 0.996 | 0.711 | 21 | 0.996664 | -0.11 | 0.013896 | 0.1523 | 0.587 | 1.155 | -0.72 | 0.212 | 0.50754 | -1 | Decrease | 0.127 | -51 |
| rs372594330 | 103 | H/Q | 0.81 | 0.013 | 0.001 | 0.001 | 0.315682 | 2.05 | 0.003194 | 0.0046 | 0.313 | -0.845 | 0.25 | 0.109 | 0.00467 | -0.32657 | Decrease | 0.155 | -73 |
| rs750686409 | 101 | Q/P | 0 | 0.994 | 0.897 | 23.2 | 0.992187 | 1.74 | 0.021782 | 0.0719 | 0.618 | 2.995 | -3.53 | 0.623 | 0.42751 | -0.89405 | Decrease | 0.741 | 56 |
| rs374248493 | 98 | R/Q | 0.12 | 0.039 | 0.263 | 11.83 | 0.9654 | -0.02 | 0.021554 | 0.0343 | - | 0.87 | -0.88 | 0.086 | 0.02219 | -0.03037 | Decrease | 0.374 | -55 |
| rs750350177 | 76 | R/K | 1 | 0.006 | 0 | 7.32 | 0.836963 | 2.03 | 0.004894 | 0.0046 | 0.392 | -0.345 | 0.42 | 0.089 | 0.06609 | -1 | Decrease | 0.088 | -58 |
| rs747377941 | 65 | S/G | 0.02 | 0.987 | 0.82 | 25.5 | 0.998442 | 1.95 | 0.026462 | 0.0788 | 0.706 | 2.545 | -2.96 | 0.839 | 0.85017 | -0.25959 | Decrease | 0.557 | 52 |
| rs374851046 | 52 | R/H | 0.01 | 0.998 | 0.88 | 34 | 0.999571 | -0.51 | 0.247638 | 0.441 | - | 3.18 | -4.21 | 0.821 | 0.62258 | -0.14326 | Decrease | 0.525 | 62 |
| rs749358844 | 52 | R/C | 0 | 0.998 | 0.919 | 34 | 0.999403 | -0.53 | 0.235127 | 0.5057 | 0.589 | 3.525 | -6.62 | 0.862 | 0.75151 | -0.41313 | Decrease | 0.639 | 74 |
| rs369181900 | 51 | C/Y | 0 | 0.998 | 0.919 | 27.9 | 0.997434 | 0.16 | 0.245604 | 0.3835 | - | 2.465 | -9.82 | 0.954 | 0.85799 | 0.7061 | Increase | 0.775 | 82 |
| rs549402254 | 50 | W/S | 0 | 0.998 | 0.919 | 29.2 | 0.990748 | -3.41 | 0.595918 | 0.9053 | 0.737 | 3.705 | -13.17 | 0.943 | 0.89191 | -1 | Decrease | 0.671 | 88 |
| rs753325601 | 47 | R/C | 0.01 | 0.998 | 0.88 | 32 | 0.999301 | -0.24 | 0.180306 | 0.3621 | 0.636 | 2.895 | -4.11 | 0.527 | 0.60996 | -0.46226 | Decrease | 0.642 | 61 |
| rs746216516 | 31 | S/F | 0.02 | 0.914 | 0.749 | 24.4 | 0.997679 | -0.42 | 0.122706 | 0.4506 | 0.637 | 3.045 | -4.45 | 0.531 | 0.82039 | 0.516677 | Increase | 0.627 | 37 |
| rs746216516 | 31 | S/Y | 0 | 0.914 | 0.81 | 24.1 | 0.99391 | -0.42 | 0.124266 | 0.4506 | - | 3.39 | -4.43 | 0.517 | 0.83687 | 1 | Increase | 0.625 | 60 |
| rs748326594 | 26 | G/A | 0.13 | 0.999 | 0.743 | 17.29 | 0.991577 | 0.01 | 0.071984 | 0.2892 | 0.781 | 1.625 | -3.18 | 0.155 | 0.35494 | -0.28298 | Decrease | 0.373 | 11 |
| rs534417680 | 20 | N/T | 0.06 | 0.995 | 0.78 | 21.5 | 0.993776 | 1.23 | 0.018702 | 0.1312 | - | 1.625 | -2.87 | 0.284 | 0.69637 | -0.14163 | Decrease | 0.76 | -60 |
| rs372634215 | 7 | L/F | 0.22 | 0.118 | 0.052 | 2.839 | 0.969995 | 0.6 | 0.013446 | 0.0581 | 0.545 | 1.65 | -1.68 | 0.161 | 0.37221 | -0.27222 | Decrease | 0.027 | -31 |
| rs374483382 | 5 | R/W | 0.34 | 0.013 | 0.024 | 5.328 | 0.557137 | 1.27 | 0.007447 | 0.0091 | - | 1.1 | -1.03 | 0.096 | 0.02424 | -0.52864 | Decrease | 0.081 | -10 |
| rs768583708 | 219 | G/C |  | 0 |  | 1.566 | 0.302039 | 0.71 | 0.001165 | 0.0267 | 0.356 | - | - | 0.072 | 0.01106 |  | Decrease |  |  |
| rs761884997 | 216 | A/V | 0.11 | 0.003 | 0.272 | 2.638 | 0.86165 | 0.99 | 0.003153 | 0.0322 | 0.213 | - | - | 0.033 | 0.0103 |  | Increase |  |  |
| rs761884997 | 216 | A/G | 0.11 | 0.113 | 0.281 | 0.688 | 0.813376 | 0.97 | 0.004473 | 0.0371 | 0.218 | - | - | 0.024 | 0.02288 |  | Decrease |  |  |
| rs767767228 | 216 | A/S | 0.22 | 0.084 | 0.048 | 2.763 | 0.479925 | 1.02 | 0.004571 | 0.0373 | 0.227 | - | - | 0.039 | 0.01883 |  | Increase |  |  |
| rs766466972 | 206 | P/S | 0.24 | 0.067 | 0.042 | 6.729 | 0.952723 | 0.9 | 0.004861 | 0.0385 | 0.404 | - | - | 0.066 | 0.0644 |  | Increase |  |  |
| rs772224354 | 184 | P/S | 0.06 | 0.563 | 0.515 | 5.818 | 0.993677 | 0.9 | 0.002991 | 0.0485 | 0.431 | - | - | 0.09 | 0.04238 |  | Decrease |  |  |
| rs766236034 | 182 | T/I | 0.29 | 0.003 | 0.029 | 2.342 | 0.937122 | 1.07 | 0.00203 | 0.0216 | 0.358 | - | - | 0.01 | 0.01253 |  | Decrease |  |  |
| rs776713120 | 182 | T/S | 0.02 | 0.058 | 0.387 | 4.802 | 0.742545 | 1.11 | 0.002719 | 0.0232 | 0.28 | - | - | 0.028 | 0.02451 |  | Decrease |  |  |
| rs765204016 | 181 | G/R | 0.01 | 0.043 | 0.408 | 1.732 | 0.721975 | 0.73 | 0.002446 | 0.0158 | 0.289 | - | - | 0.058 | 0.01548 |  | Increase |  |  |
| rs763760706 | 173 | K/R | 0.51 | 0.001 | 0.009 | 1.391 | 0.865052 | 1.04 | 0.001118 | 0.0133 | 0.166 | - | - | 0.029 | 0.00046 |  | Decrease |  |  |
| rs756478866 | 169 | D/E | 0.91 | 0 | 0 | 1.221 | 0.735829 | 0.98 | 0.001035 | 0.0262 | 0.187 | - | - | 0.015 | 0.00237 |  | Decrease |  |  |
| rs758556018 | 221 | L/F | 0 | 0.844 | 0.768 | 25.9 | 0.998652 | 0.6 | 0.013355 | 0.2038 | 0.469 | 1.935 | -1.52 | 0.403 | 0.84645 | -1 | Decrease | 0.317 | 33 |
| rs777941887 | 218 | G/A | 0.01 | 0.593 | 0.611 | 9.02 | 0.986289 | 0.99 | 0.003611 | 0.0553 | 0.7 | 2.085 | -0.88 | 0.292 | 0.03554 | -0.59464 | Decrease | 0.539 | -3 |
| rs769593356 | 204 | G/E | 0.44 | 0 | 0.013 | 0.014 | 0.526263 | 1.01 | 0.004373 | 0.0196 | 0.178 | - | -0.78 | 0.048 | 0.00562 | 0.327594 | Increase | 0.154 | -4 |
| rs762894308 | 199 | H/D | 0.41 | 0.034 | 0.017 | 8.103 | 0.92773 | 1.34 | 0.005448 | - | - | - | - | 0.072 | 0.36113 | 0.807726 | Increase | 0.381 | 64 |
| rs762894308 | 212 | A/G | 0.7 | 0.018 | 0.003 | 8.103 | 0.92773 | 1.34 | 0.005448 | - | - | - | - | 0.072 | 0.36113 |  | Increase |  |  |
| rs768572417 | 197 | A/V | 0.05 | 0.196 | 0.364 | 27.6 | 0.997499 | 0.6 | 0.007079 | 0.0891 | 0.483 | - | -0.93 | 0.303 | 0.86203 | 0.221911 | Increase | 0.3 | 26 |
| rs773918521 | 195 | A/V | 0.24 | 0 | 0.037 | 1.309 | 0.583792 | 1.03 | 0.002485 | 0.0199 | 0.533 | - | -0.27 | 0.171 | 0.15014 | 1 | Decrease | 0.36 | -85 |
| rs761283027 | 193 | L/F | 0 | 0.031 | 0.446 | 25.1 | 0.996148 | 0.3 | 0.065365 | 0.1028 | 0.679 | - | -1.92 | 0.346 | 0.77924 | -1 | Decrease | 0.352 | 47 |
| rs761283027 | 206 | P/L | 0 | 0.675 | 0.684 | 25.1 | 0.996148 | 0.3 | 0.065365 | 0.1028 | 0.679 | - | -1.92 | 0.346 | 0.77924 |  | Increase |  |  |
| rs765569948 | 191 | S/T | 0.21 | 0.098 | 0.051 | 0.164 | 0.617254 | 1.01 | 0.006135 | - | - | - | - | 0.053 | 0.06086 | 0.660339 | Increase | 0.362 | -5 |
| rs765569948 | 191 | S/N | 0.08 | 0.107 | 0.308 | 3.531 | 0.929387 | 0.88 | 0.005329 | 0.0492 | 0.5 | - | -1 | 0.097 | 0.0469 | 0.942443 | Increase | 0.676 | -46 |
| rs765569948 | 204 | Q/H | 0.01 | 0.005 | 0.406 | 0.164 | 0.617254 | 1.01 | 0.006135 | - | - | - | - | 0.053 | 0.06086 |  | Decrease |  |  |
| rs778415288 | 181 | C/W | 0.01 | 0.97 | 0.818 | 27.3 | 0.994116 | 0.68 | 0.032744 | 0.1507 | 0.674 | - | -5.42 | 0.722 | 0.57859 | -0.86207 | Decrease | 0.873 | 70 |
| rs778415288 | 195 | H/D | 0.28 | 0.7 | 0.283 | 27.3 | 0.994116 | 0.68 | 0.032744 | 0.1507 | 0.674 | - | -5.42 | 0.722 | 0.57859 |  | Decrease |  |  |
| rs778415288 | 195 | H/N | 0.44 | 0.506 | 0.182 | 39 | 0.993945 | - | - | - | - | - | - | - | 0.48408 |  | Decrease |  |  |
| rs757387879 | 178 | L/R | 0 | 0.97 | 0.857 | 29 | 0.997621 | 0.33 | 0.025879 | 0.2541 | 0.733 | - | -2.25 | 0.815 | 0.87576 | -0.52151 | Decrease | 0.699 | 55 |
| rs764489173 | 158 | S/R | 0 | 0.994 | 0.897 | 25.8 | 0.998154 | 0.63 | 0.054991 | 0.2527 | 0.477 | 2.33 | -2.9 | 0.571 | 0.82845 | 0.419974 | Increase | 0.506 | 37 |
| rs766647311 | 145 | G/W | 0.01 | 0.965 | 0.812 | 26.1 | 0.992926 | 1.26 | 0.024322 | 0.0773 | 0.473 | 0.895 | -1.32 | 0.32 | 0.0941 | 1 | Decrease | 0.347 | 32 |
| rs766647311 | 145 | G/R | 0.07 | 0.281 | 0.405 | 16.31 | 0.980879 | 1.32 | 0.013011 | 0.0197 | 0.354 | 0.895 | -0.13 | 0.249 | 0.05161 | 1 | Increase | 0.327 | -5 |
| rs778620014 | 142 | W/R | 0 | 0.998 | 0.919 | 24.6 | 0.993682 | 0.94 | 0.018312 | 0.1256 | 0.668 | 2.33 | -4.44 | 0.73 | 0.50249 | -0.87289 | Decrease | 0.708 | 68 |
| rs758428150 | 137 | D/N | 0.09 | 0.503 | 0.465 | 7.974 | 0.991717 | 1.15 | 0.005094 | 0.0427 | 0.61 | 1.935 | -0.17 | 0.027 | 0.09452 |  | Decrease | 0.564 | -32 |
| rs772641807 | 136 | R/W | 0.01 | 0.648 | 0.633 | 22.5 | 0.993001 | 1.17 | 0.013309 | 0.04 | 0.448 | - | -1.55 | 0.162 | 0.31379 | -0.8573 | Decrease | 0.434 | 47 |
| rs762052858 | 131 | D/G | 0.1 | 0.942 | 0.673 | 23 | 0.996378 | 0.86 | - | 0.1529 | - | 2.295 | -1.79 | 0.348 | 0.91016 | -1 | Decrease | 0.656 | 35 |
| rs759576705 | 130 | A/T | 0.25 | 0.029 | 0.038 | 12.28 | 0.968565 | 1.29 | 0.003917 | 0.0305 | 0.411 | 1.43 | -0.18 | 0.034 | 0.02624 | -1 | Decrease | 0.312 | -68 |
| rs757209611 | 115 | G/S | 0.26 | 0.999 | 0.524 | 23.6 | 0.99791 | -0.01 | 0.014343 | 0.1491 | 0.533 | 0.54 | -0.94 | 0.502 | 0.04132 | 0.096259 | Increase | 0.337 | -52 |
| rs755641572 | 111 | Q/K | 0.2 | 0.979 | 0.471 | 23.3 | 0.992006 | -0.14 | 0.033968 | 0.1954 | 0.641 | 1.7 | -1.36 | 0.433 | 0.70762 | 0.615453 | Increase | 0.326 | -19 |
| rs775784725 | 86 | D/G | 0 | 0.998 | 0.919 | 23.7 | 0.996834 | -0.07 | 0.304303 | 0.4226 | 0.726 | 3.59 | -6.46 | 0.948 | 0.74041 | -1 | Decrease | 0.74 | 43 |
| rs764373520 | 83 | A/T | 0.08 | 0.54 | 0.483 | 27.1 | 0.999091 | 2.26 | 0.008873 | 0.0193 | 0.319 | 1.16 | -1.23 | 0.238 | 0.13463 | 0.064883 | Increase | 0.387 | 32 |
| rs774054773 | 80 | G/R | 0.02 | 1 | 0.883 | 30 | 0.999356 | 1.45 | 0.123967 | 0.1797 | - | 3.245 | -5.94 | 0.929 | 0.78432 | -0.30439 | Decrease | 0.773 | 72 |
| rs755692913 | 75 | L/Q | 0.19 | 0.999 | 0.54 | 24 | 0.995448 | -0.07 | 0.045073 | 0.1833 | 0.566 | 1.985 | -1.42 | 0.689 | 0.899 | -1 | Decrease | 0.804 | 38 |
| rs765933093 | 72 | L/V | 0.04 | 0.996 | 0.81 | 24.6 | 0.99828 | 1.97 | 0.006146 | 0.0439 | - | 1.87 | -0.83 | 0.237 | 0.75339 | -0.25484 | Decrease | 0.598 | -5 |
| rs767357967 | 49 | A/S | 0.29 | 0.996 | 0.491 | 22.8 | 0.995507 | -0.04 | 0.027927 | 0.1445 | 0.591 | 0.855 | -0.68 | 0.211 | 0.57413 | -0.69249 | Decrease | 0.099 | -19 |
| rs767357967 | 49 | A/T | 0.14 | 0.998 | 0.724 | 23.7 | 0.999234 | -0.14 | 0.014304 | 0.1614 | 0.55 | 1.485 | -1.46 | 0.22 | 0.44692 | 0.118146 | Increase | 0.203 | 5 |
| rs757622796 | 35 | S/P | 0.05 | 0.997 | 0.811 | 23.1 | 0.997472 | 1.7 | 0.018472 | 0.0736 | 0.532 | 2.57 | -2.69 | 0.654 | 0.69499 | 0.019446 | Increase | 0.737 | -9 |
| rs757622796 | 35 | S/T | 0.12 | 0.991 | 0.705 | 18.73 | 0.989284 | 1.78 | 0.012892 | 0.0518 | 0.516 | 1.675 | -1.23 | 0.304 | 0.56085 | -0.03461 | Decrease | 0.508 | -56 |
| rs768745050 | 27 | V/L | 0.11 | 0.03 | 0.273 | 5.813 | 0.886325 | -0.21 | 0.011793 | 0.0646 | 0.544 | 0.91 | -1.28 | 0.107 | 0.02143 | -0.29704 | Decrease | 0.252 | -35 |
| rs768745050 | 27 | V/M | 0.54 | 0.053 | 0.011 | 0.912 | 0.612003 | -0.25 | 0.01796 | 0.0378 | 0.556 | 0.605 | -0.58 | 0.064 | 0.00608 | -0.28628 | Decrease | 0.059 | -76 |
| rs772412391 | 25 | Q/R | 0.08 | 0.038 | 0.302 | 0.127 | 0.729698 | -0.09 | 0.019543 | 0.0547 | 0.593 | 1.645 | -1.93 | 0.125 | 0.51586 | 0.17551 | Increase | 0.455 | -45 |
| rs760511786 | 22 | T/I | 0 | 0.943 | 0.832 | 23.4 | 0.995774 | -0.21 | 0.090371 | 0.3441 | 0.68 | 2.81 | -4.23 | 0.534 | 0.79687 | -0.91393 | Increase | 0.532 | 18 |
| rs760511786 | 22 | T/K | 0.01 | 0.88 | 0.749 | 23.9 | 0.980997 | -0.03 | 0.075812 | 0.2321 | 0.694 | 1.915 | -3.12 | 0.629 | 0.8266 | -0.60893 | Decrease | 0.607 | 34 |
| rs776578102 | 22 | T/S | 0.12 | 0.203 | 0.28 | 15.87 | 0.929379 | -0.06 | 0.051367 | 0.0728 | 0.667 | 0.935 | -1.76 | 0.311 | 0.6781 | -0.95892 | Decrease | 0.273 | -34 |
| rs776578102 | 22 | T/A | 0.03 | 0.64 | 0.589 | 22.6 | 0.991997 | -0.14 | 0.057253 | 0.1759 | 0.671 | 2.26 | -2.89 | 0.419 | 0.64323 | -1 | Decrease | 0.381 | 26 |
| rs764738032 | 19 | H/Y | 0.1 | 0.018 | 0.287 | 0.002 | 0.793266 | 1.28 | 0.003498 | 0.0391 | 0.345 | 1.3 | -2.41 | 0.12 | 0.38278 | -0.04526 | Increase | 0.538 | -50 |
| rs764738032 | 19 | H/N | 0.03 | 0.592 | 0.569 | 11.77 | 0.925755 | 1.34 | 0.006999 | 0.058 | 0.318 | 1.445 | -2.03 | 0.101 | 0.55204 | -0.07946 | Decrease | 0.491 | -14 |
| rs777808487 | 16 | S/F | 0 | 0.998 | 0.919 | 21.5 | 0.994255 | 1.12 | 0.022767 | 0.1028 | 0.581 | 2.35 | -3.28 | 0.355 | 0.10607 | -0.2537 | Increase | 0.737 | 2 |
| rs1232354245 | 219 | G/D |  | 0.001 |  | 0.363 | 0.219423 | 0.75 | 0.001453 | 0.0286 | 0.32 | - | - | 0.074 | 0.01975 |  | Decrease |  |  |
| rs1287533624 | 218 | T/I | 0.01 | 0 | 0.406 | 0.626 | 0.559961 | 1.07 | 0.001655 | 0.0272 | 0.21 | - | - | 0.02 | 0.01153 |  | Increase |  |  |
| rs924875210 | 217 | G/R | 0 | 0.001 | 0.445 | 1.022 | 0.605221 | 0.73 | 0.002304 | 0.0206 | 0.255 | - | - | 0.073 | 0.00929 |  | Decrease |  |  |
| rs912186779 | 208 | H/L | 0.31 | 0 | 0.027 | 5.849 | 0.744142 | 1.08 | 0.003951 | 0.0268 | 0.435 | - | - | 0.034 | 0.00856 |  | Decrease |  |  |
| rs947954226 | 193 | P/L | 0 | 0.714 | 0.703 | 5.492 | 0.99447 | 0.87 | 0.010209 | 0.0939 | 0.53 | - | - | 0.11 | 0.05671 |  | Decrease |  |  |
| rs1268616215 | 189 | A/T | 0.02 | 0.011 | 0.384 | 1.67 | 0.739739 | 0.99 | 0.004382 | 0.0222 | 0.312 | - | - | 0.014 | 0.00442 | -1 | Decrease | 0.377 | 11 |
| rs1005368749 | 188 | L/F | 0.09 | 0.003 | 0.296 | 4.27 | 0.951366 | 0.84 | 0.004201 | 0.0236 | 0.31 | - | - | 0.025 | 0.01627 | -1 | Decrease | 0.514 | 48 |
| rs1169415398 | 167 | K/R | 1 | 0 | 0 | 7.216 | 0.499243 | 1.04 | 0.005915 | 0.0151 | 0.21 | - | - | 0.031 | 0.00105 |  | Decrease |  |  |
| rs1017615278 | 166 | V/G | 0 | 0.466 | 0.598 | 5.065 | 0.640639 | 0.93 | - | 0.1438 | 0.19 | - | -0.1 | - | 0.8835 |  | Decrease |  |  |
| rs779444937 | 162 | A/T | 0.04 | 0.025 | 0.35 | 7.877 | 0.980871 | 1.11 | 0.005768 | 0.0282 | 0.27 | - | 0.7 | - | 0.034 |  |  |  |  |
| rs1205300439 | 225 | P/L | 0.01 | 0.007 | 0.406 | 23.4 | 0.970004 | 1.08 | 0.004168 | 0.0449 | 0.2 | 0.69 | -1.31 | 0.062 | 0.86667 | 0.129187 | Increase | 0.133 | 23 |
| rs1276123772 | 225 | P/S | 0.12 | 0 | 0.261 | 4.481 | 0.627446 | 1.21 | 0.004778 | 0.0151 | 0.103 | 0.69 | 0.37 | 0.043 | 0.25631 | -0.90825 | Decrease | 0.092 | -19 |
| rs1161481912 | 215 | H/Q | 0.24 | 0.007 | 0.037 | 0.031 | 0.75248 | 1 | 0.002426 | 0.0198 | - | 1.445 | -0.72 | 0.024 | 0.01208 | -1 | Decrease | 0.071 | -30 |
| rs1170630105 | 212 | D/E | 0.1 | 0.012 | 0.287 | 2.083 | 0.814991 | 0.85 | 0.003355 | 0.0355 | 0.178 | 2.005 | -0.23 | 0.055 | 0.01987 | -0.28461 | Increase | 0.421 | 13 |
| rs1279995205 | 194 | W/R | 0.02 | 0.564 | 0.576 | 25.7 | 0.99062 | 0.72 | 0.053048 | - | - | - | - | 0.092 | 0.81563 | 0.93713 | Increase | 0.667 | 76 |
| rs1279995205 | 207 | L/P | 0 | 0.986 | 0.879 | 25.7 | 0.99062 | 0.72 | 0.053048 | - | - | - | - | 0.092 | 0.81563 |  | Decrease |  |  |
| rs1173193141 | 187 | I/V | 0.06 | 0.003 | 0.323 | 1.829 | 0.779635 | 1.24 | 0.009871 | - | - | - | - | 0.255 | 0.33285 | -0.36805 | Decrease | 0.154 | -7 |
| rs1173193141 | 200 | D/G | 0.43 | 0.007 | 0.013 | 1.829 | 0.779635 | 1.24 | 0.009871 | - | - | - | - | 0.255 | 0.33285 |  | Decrease |  |  |
| rs1306444285 | 176 | L/F | 0.42 | 0.052 | 0.017 | 18.51 | 0.988923 | 0.56 | 0.009559 | 0.0637 | 0.562 | - | -1.39 | 0.265 | 0.04541 |  | Decrease | 0.289 | -15 |
| rs1306444285 | 189 | P/L | 0.06 | 0.005 | 0.323 | 18.51 | 0.988923 | 0.56 | 0.009559 | 0.0637 | 0.562 | - | -1.39 | 0.265 | 0.04541 |  | Decrease |  |  |
| rs1274963337 | 167 | E/K | 0.08 | 0.003 | 0.3 | 12.52 | 0.88982 | 1.01 | 0.011568 | 0.0301 | 0.451 | - | -0.92 | 0.188 | 0.02158 |  | Decrease | 0.188 | 2 |
| rs1274963337 | 180 | R/K | 0.46 | 0.003 | 0.012 | 12.52 | 0.88982 | 1.01 | 0.011568 | 0.0301 | 0.451 | - | -0.92 | 0.188 | 0.02158 |  | Decrease |  |  |
| rs779960537 | 166 | G/E | 1 | 0 | 0 | 0.015 | 0.556599 | 1.19 | 0.00288 | 0.0048 | 0.304 | - | -0.29 | 0.172 | 0.00564 | 0.198208 | Increase | 0.064 | -37 |
| rs1275430164 | 160 | S/F | 0 | 0.996 | 0.906 | 25.8 | 0.997717 | 1 | 0.038164 | 0.3012 | 0.504 | 2.295 | -2.49 | 0.313 | 0.91762 | -0.57295 | Decrease | 0.478 | 52 |
| rs1255249948 | 157 | H/R | 0.19 | 0.268 | 0.078 | 0.263 | 0.954478 | 1.18 | 0.005224 | 0.0351 | 0.33 | 1.5 | -1.97 | 0.036 | 0.51235 | 0.353519 | Increase | 0.346 | 20 |
| rs1187953302 | 153 | A/V | 0.44 | 0.009 | 0.013 | 0.012 | 0.883982 | 1.08 | 0.007352 | 0.0176 | 0.453 | 0.425 | -0.67 | 0.076 | 0.01904 | -1 | Decrease | 0.103 | -80 |
| rs867253281 | 149 | S/N | 0.07 | 0.498 | 0.486 | 15.46 | 0.98724 | 1.07 | 0.009307 | 0.0799 | 0.441 | 2.175 | -0.72 | 0.081 | 0.03685 | -0.08173 | Increase | 0.398 | 0 |
| rs1257994892 | 132 | P/T | 0.07 | 0.916 | 0.686 | 17.62 | 0.991554 | 1.08 | 0.020004 | 0.1314 | 0.57 | 2.255 | -3.11 | 0.371 | 0.86954 | -0.95921 | Decrease | 0.43 | -42 |
| rs781244560 | 114 | H/Q | 0.22 | 0.033 | 0.044 | 0.125 | 0.879467 | -0.09 | 0.015207 | 0.0258 | 0.34 | 0.365 | -1.37 | 0.028 | 0.00848 |  | Decrease | 0.203 | -2 |
| rs779888024 | 108 | Y/C | 0 | 0.999 | 0.935 | 24.8 | 0.998226 | -3.44 | 0.438915 | 0.911 | 0.683 | 3.73 | -9 | 0.919 | 0.85017 | 0.04364 | Increase | 0.625 | 78 |
| rs1173985669 | 102 | P/T | 0.27 | 0.022 | 0.035 | 0.005 | 0.753893 | -0.2 | 0.013621 | 0.0444 | 0.342 | 0.895 | 0.26 | 0.105 | 0.01071 | -1 | Decrease | 0.085 | -59 |
| rs1204617414 | 93 | L/R | 0 | 0.974 | 0.861 | 24.7 | 0.997264 | -0.35 | 0.129571 | 0.3936 | 0.707 | 3.04 | -4.51 | 0.922 | 0.90405 | -0.45626 | Decrease | 0.662 | 70 |
| rs914179215 | 93 | L/V | 0.27 | 0.203 | 0.053 | 8.47 | 0.95744 | -0.15 | 0.02714 | 0.0866 | 0.545 | 1.295 | -1.28 | 0.299 | 0.75692 | 0.106897 | Decrease | 0.056 | -14 |
| rs1271841898 | 90 | G/D | 0.18 | 0.982 | 0.482 | 22.4 | 0.99572 | -0.12 | 0.081543 | 0.1345 | 0.262 | 1.55 | -1.12 | 0.26 | 0.15374 | -0.68346 | Decrease | 0.374 | -17 |
| rs1018859599 | 68 | N/S | 0.83 | 0.01 | 0.001 | 0.011 | 0.688776 | -0.09 | 0.007659 | 0.0364 | 0.342 | -0.715 | 0.12 | 0.021 | 0.00531 | -0.11705 | Decrease | 0.186 | -62 |
| rs1229671015 | 67 | H/Q | 0.07 | 0.996 | 0.78 | 22.2 | 0.990301 | -0.09 | 0.022892 | 0.1183 | 0.623 | 1 | -1.64 | 0.299 | 0.3152 | 0.637869 | Increase | 0.659 | 5 |
| rs1247550853 | 59 | P/L | 0.29 | 0.022 | 0.03 | 8.709 | 0.792467 | -0.29 | 0.0096 | 0.0391 | 0.509 | 0.93 | -1.61 | 0.102 | 0.01666 | 0.030896 | Increase | 0.208 | 0 |
| rs954383723 | 54 | L/Q | 0.19 | 0.885 | 0.395 | 17.46 | 0.990832 | -0.12 | 0.017197 | 0.1458 | 0.568 | 1.525 | -0.4 | 0.404 | 0.51826 | -1 | Decrease | 0.517 | 0 |
| rs1299147476 | 45 | G/W | 0.32 | 0.998 | 0.498 | 24.6 | 0.994026 | 1.95 | 0.015408 | 0.0551 | 0.581 | 1.4 | -0.7 | 0.352 | 0.14051 | 0.246118 | Increase | 0.468 | -1 |
| rs1189666053 | 40 | S/P | 0.61 | 0.041 | 0.007 | 13.28 | 0.982142 | -0.36 | 0.012348 | 0.0351 | 0.508 | 0.625 | 0.91 | 0.156 | 0.24414 | -0.4957 | Decrease | 0.445 | -39 |
| rs1279888023 | 39 | D/A | 0.01 | 0.797 | 0.705 | 26.1 | 0.99573 | 1.96 | 0.01088 | 0.0451 | 0.489 | 1.78 | -3.55 | 0.387 | 0.76875 | 0.187039 | Increase | 0.468 | 20 |
| rs937164872 | 38 | Y/H | 0 | 0.998 | 0.919 | 25.3 | 0.998148 | -0.34 | 0.162666 | 0.4837 | - | 3.31 | -4.83 | 0.821 | 0.89693 | -0.99873 | Decrease | 0.649 | 88 |
| rs1294900634 | 23 | V/L | 0.04 | 0.99 | 0.79 | 22.7 | 0.995924 | -0.13 | 0.060988 | 0.2221 | 0.574 | 1.935 | -2.04 | 0.182 | 0.07898 | 0.846284 | Decrease | 0.489 | 31 |
| rs1249745636 | 12 | V/A | 0.17 | 0.013 | 0.059 | 0.017 | 0.267536 | 1.15 | 0.00689 | 0.0407 | 0.474 | 2.24 | -1.76 | 0.095 | 0.1803 | -1 | Decrease | 0.24 | -8 |
| rs1212795515 | 12 | V/L | 0.24 | 0.146 | 0.05 | 2.944 | 0.885534 | 1.32 | 0.00726 | 0.0195 | 0.403 | 1.89 | -0.39 | 0.078 | 0.03626 | 0.371725 | Increase | 0.381 | -72 |
| rs1187834290 | 2 | E/D | 0.15 | 0.015 | 0.241 | 1.15 | 0.990334 | 1.13 | 0.005235 | 0.0335 | 0.224 | 1.5 | -1.18 | 0.13 | 0.1097 | 0.011971 | Decrease | 0.095 | -88 |
| rs1484828242 | 215 | E/K | 0 | 0.058 | 0.448 | 5.479 | 0.958552 | 1 | 0.006773 | 0.0328 | 0.196 | - | - | 0.149 | 0.03244 |  | Decrease |  |  |
| rs1451021840 | 210 | F/L | 0 | 0.003 | 0.445 | 0.441 | 0.888275 | 1.11 | 0.004995 | 0.0283 | 0.387 | - | - | 0.025 | 0.02398 |  | Decrease |  |  |
| rs1391283629 | 200 | W/C | 0 | 0.865 | 0.78 | 4.833 | 0.825626 | 0.88 | 0.009521 | 0.0942 | 0.63 | - | - | 0.223 | 0.054 |  | Decrease |  |  |
| rs1439075848 | 199 | L/P | 0 | 0.018 | 0.445 | 3.858 | 0.843228 | 0.8 | 0.005048 | 0.0297 | 0.57 | - | - | 0.134 | 0.1907 |  | Increase |  |  |
| rs1414757369 | 178 | V/L | 0.31 | 0.012 | 0.028 | 4.917 | 0.856393 | 1.06 | 0.002035 | 0.0217 | 0.132 | - | - | 0.033 | 0.08831 |  | Decrease |  |  |
| rs1333444417 | 209 | S/N | 0.1 | 0.074 | 0.291 | 2.174 | 0.85642 | 0.96 | 0.002615 | 0.0336 | 0.143 | - | -0.9 | 0.044 | 0.02511 | -0.46113 | Decrease | 0.222 | -7 |
| rs1360894001 | 199 | H/R | 0.99 | 0 | 0 | 0.001 | 0.800274 | 1.13 | 0.001912 | 0.0221 | 0.3 | - | -0.07 | 0.02 | 0.00428 | 1 | Increase | 0.094 | -21 |
| rs1345120258 | 196 | A/T | 0.11 | 0.014 | 0.272 | 14.66 | 0.870555 | 1.04 | 0.005775 | 0.0443 | 0.417 | - | -0.28 | 0.211 | 0.14887 | -1 | Decrease | 0.233 | -72 |
| rs1345120258 | 209 | C/Y | 0 | 0.837 | 0.764 | 14.66 | 0.870555 | 1.04 | 0.005775 | 0.0443 | 0.417 | - | -0.28 | 0.211 | 0.14887 |  | Increase |  |  |
| rs1489281880 | 195 | A/S | 0.08 | 0 | 0.3 | 19.65 | 0.9745 | 0.93 | 0.015934 | 0.0579 | 0.594 | - | -0.91 | 0.235 | 0.43074 | -0.52485 | Decrease | 0.441 | -65 |
| rs1489281880 | 208 | G/V | 0 | 0.17 | 0.46 | 19.65 | 0.9745 | 0.93 | 0.015934 | 0.0579 | 0.594 | - | -0.91 | 0.235 | 0.43074 |  | Decrease |  |  |
| rs1425834660 | 184 | L/P | 0.01 | 0.879 | 0.748 | 26.3 | 0.998607 | 0.06 | 0.045822 | 0.268 | 0.678 | - | -1.97 | 0.92 | 0.81521 | -0.97965 | Decrease | 0.754 | 72 |
| rs1368290241 | 178 | L/F | 0.04 | 0.271 | 0.378 | 24.3 | 0.994921 | 0.46 | 0.027227 | 0.0694 | 0.672 | - | -1.53 | 0.452 | 0.72148 | -0.85433 | Decrease | 0.281 | -55 |
| rs1368290241 | 191 | P/L | 0.05 | 0.593 | 0.55 | 24.3 | 0.994921 | 0.46 | 0.027227 | 0.0694 | 0.672 | - | -1.53 | 0.452 | 0.72148 |  | Increase |  |  |
| rs1430713564 | 146 | E/Q | 0.1 | 0.082 | 0.292 | 11.13 | 0.975891 | 1.06 | 0.00654 | 0.0453 | 0.406 | 1.195 | -1.13 | 0.107 | 0.1173 | -0.10331 | Decrease | 0.245 | -24 |
| rs1344427098 | 135 | H/D | 0.24 | 0.001 | 0.037 | 0.012 | 0.84773 | 1.48 | 0.004766 | 0.0191 | 0.301 | 1.43 | 0.02 | 0.066 | 0.0491 | -0.27865 | Decrease | 0.276 | -13 |
| rs1482867735 | 133 | L/M | 0.05 | 0.914 | 0.712 | 15.26 | 0.99181 | 1.03 | 0.011426 | 0.1039 | 0.547 | 2.045 | -0.28 | 0.163 | 0.10758 | -0.87261 | Decrease | 0.35 | -65 |
| rs1409131974 | 104 | D/G | 0 | 0.998 | 0.919 | 23.6 | 0.996001 | -2.33 | 0.335989 | 0.7907 | 0.678 | 3.73 | -6.8 | 0.903 | 0.74126 | -0.4373 | Decrease | 0.683 | 85 |
| rs1311610930 | 97 | L/P | 0 | 0.999 | 0.935 | 24.1 | 0.998286 | -0.35 | 0.249112 | 0.4364 | 0.697 | 3.135 | -5.62 | 0.911 | 0.70465 | -1 | Decrease | 0.707 | 58 |
| rs1483052893 | 88 | T/I | 0.38 | 0.018 | 0.018 | 1.758 | 0.316209 | -0.15 | 0.016551 | 0.0662 | 0.476 | 0.435 | -1.45 | 0.096 | 0.15934 | -1 | Decrease | 0.254 | -69 |
| rs1425755566 | 82 | T/S | 0.03 | 0.994 | 0.817 | 23.8 | 0.995871 | 1.98 | 0.021815 | 0.0715 | 0.799 | 2.105 | -2.25 | 0.662 | 0.86627 | 0.833518 | Increase | 0.627 | 17 |
| rs1432256574 | 81 | S/N | 0.07 | 0.994 | 0.771 | 26.7 | 0.997133 | 1.97 | 0.035242 | 0.097 | 0.556 | 3.005 | -1.23 | 0.292 | 0.59831 | 0.085456 | Increase | 0.629 | 55 |
| rs1379518570 | 68 | N/Y | 0.56 | 0.689 | 0.253 | 11.27 | 0.984741 | -0.22 | 0.023847 | 0.113 | 0.416 | 0.64 | -1.82 | 0.143 | 0.0494 | 0.763081 | Increase | 0.412 | -34 |
| rs1404747261 | 67 | H/Y | 0.05 | 0.994 | 0.797 | 23.3 | 0.997816 | -0.11 | 0.048231 | 0.2895 | 0.607 | 1.65 | -2.8 | 0.364 | 0.80355 | 1 | Increase | 0.668 | -13 |
| rs1389735521 | 57 | K/E | 1 | 0.006 | 0 | 0.002 | 0.587835 | 1.74 | 0.007512 | 0.0052 | 0.42 | -0.995 | 1.19 | 0.074 | 0.00326 | 0.59261 | Increase | 0.069 | -25 |
| rs1359529169 | 49 | A/V | 0.08 | 0.997 | 0.766 | 24 | 0.99926 | -0.18 | 0.029361 | 0.1793 | 0.586 | 1.25 | -1.93 | 0.221 | 0.6834 | 0.309003 | Decrease | 0.215 | 19 |
| rs1435233370 | 46 | R/K | 0.15 | 0.987 | 0.677 | 20.8 | 0.994515 | 1.99 | 0.010943 | 0.0414 | 0.708 | 1.095 | -0.95 | 0.235 | 0.0508 | -1 | Decrease | 0.542 | 28 |
| rs1332526817 | 43 | H/R | 0.04 | 0.173 | 0.365 | 24 | 0.990621 | 2.01 | 0.007832 | 0.0315 | 0.588 | 2.215 | -3.46 | 0.337 | 0.84993 | -0.12422 | Decrease | 0.599 | 64 |
| rs1395807649 | 41 | M/T | 0.67 | 0.057 | 0.006 | 0.043 | 0.79298 | 2.05 | 0.004825 | 0.005 | 0.442 | -0.125 | -0.21 | 0.088 | 0.01182 | -1 | Decrease | 0.396 | -10 |
| rs1451130693 | 16 | S/P | 0 | 0.997 | 0.911 | 22.8 | 0.989244 | 1.14 | 0.027716 | 0.1049 | 0.568 | 2.35 | -2.14 | 0.546 | 0.17233 | -0.31609 | Increase | 0.814 | -3 |
| rs2234253 | 96 | T/K | 0 | 0.998 | 0.5 | 24.3 | 0.977785 | -0.18 | - | 0.3936 | - | 3.12 | -4.49 | 0.35 | 0.8697 | 0.8 | Decrease | 0.44 | 67 |
| rs75932628 | 47 | R/H | 0.8 | 0.998 | 0.33 | 26.1 | 0.99954 | -0.21 | 0.079317 | 0.277 | - | 2.895 | -2.56 | 0.397 | 0.44466 | 0.8 | Decrease | 0.12 | 35 |
