# Supplementary material for: Dynamic insights into the effects of nonsynonymous polymorphisms (nsSNPs) on loss of TREM2 function
Source: Sci Rep. 2022 Jun 7;12:9378. doi: 10.1038/s41598-022-13120-5 (PMC9174165; doi:10.1038/s41598-022-13120-5)
Supplement: Supplementary file 6 — Supplementary Information 2. [file 41598_2022_13120_MOESM6_ESM.doc]

**Supplementary File 2**

**Dynamic insights into the effects of nonsynonymous polymorphisms (nsSNPs) on loss of TREM2 function**

Raju Dash^1^, Yeasmin Akter Munni^1^, Sarmistha Mitra^1^, Ho Jin Choi^1^, Sultana Israt Jahan^2^, Apusi Chowdhury^3^, Tae Jung Jang^4^, Il Soo Moon^1^

^1^Department of Anatomy, Dongguk University College of Medicine, Gyeongju, 38066, Republic of Korea

^2^Department of Biotechnology and Genetic Engineering, Noakhali Science and Technology University, Noakhali, 3814, Bangladesh.

^3^Department of Pharmaceutical Science, North-South University, Dhaka-12 29, Bangladesh

^4^Department of Pathology, Dongguk University College of Medicine, Gyeongju 38066, Republic of Korea

* Correspondence:

Il Soo Moon

Email: [moonis@dongguk.ac.kr](mailto:moonis@dongguk.ac.kr)

**Table S1.** Prediction cut-offs used to identify deleterious SNPs in the *TREM2* gene.

| SL.NO | Name of tool | Prediction method | Cut-off value |
| --- | --- | --- | --- |
| 1. | SIFT | Sequence | ≤0.05 |
| 2. | Polyphen | Sequence and structure | ˃0.9 |
| 3. | Condel | Sequence | >0.9 |
| 4. | CADD | Sequence | >20 |
| 5. | PROVEAN | Sequence | ≤−2.5 |
| 6. | DANN | Sequence | >0.5 |
| 7. | FATHMM | Sequence | >0.5 |
| 9. | M-CAP | Sequence | >0.025 |
| 10. | MetaLR | Sequence | >0.5 |
| 11. | MutPred | Sequence and structure | >0.75 |
| 12. | MutationAssessor | Sequence and structure | >2 |
| 13. | VEST3 | Sequence | <0.05 |
| 14. | fathmm-MKL | Sequence | >0.5 |
| 15. | MuPro | Sequence | >0.5 |
| 16. | iStable | Sequence and Structure | Decrease |
| 17. | PhD-SNP | Sequence | >0.5 |
| 18. | SNAP2 | Sequence and structure | >50 |

**Table S2.** Cumulative predictions of deleterious nsSNPs in the TREM2 gene.

| RS ID | Substitution | SIFT | Polyphen | Condel | CADD | PROVEAN | DANN | FATHMM | M-CAP | MetaLR | MutPred | MutationAssessor | VEST3 | fathmm-MKL | MuPro | iStable | PhD-SNP | SNAP-2 |
| --- | --- | --- | --- | --- | --- | --- | --- | --- | --- | --- | --- | --- | --- | --- | --- | --- | --- | --- |
| rs549402254 | W50S | **0** | **0.99** | **0.91** | **29.2** | **-13.17** | **0.99** | -3.41 | **0.60** | **0.90** | **0.73** | **3.70** | 0.94 | **0.89** | **-1** | **Decrease** | **0.67** | **88** |
| rs749358844 | R52C | **0** | **0.99** | **0.91** | **34** | **-6.62** | **0.99** | -0.53 | **0.24** | **0.50** | **0.58** | **3.52** | 0.86 | **0.75** | **-0.41** | **Decrease** | **0.63** | **74** |
| rs1409131974 | D104G | **0** | **0.99** | **0.91** | **23.6** | **-6.8** | **0.99** | -2.33 | **0.34** | **0.79** | **0.67** | **3.7** | 0.90 | **0.74** | **-0.43** | **Decrease** | **0.68** | **85** |

Bold font indicates each tool indicated the shown SNP is deleterious, that is, the following criteria were satisfied SIFT (≤0.05), Polyphen ˃0.9, Condel (>0.5), CADD (>15>20), PROVEAN (≤−2.5), DANN (>0.5), FATHMM (>0.5), M-CAP (>0.025), MetaLR (>0.5), MutPred (>0.5), MutationAssessor (>2), fathmm-MKL >0.5, iStable Score (Decrease), MuPro (>0.5), PhD-SNP (<0.5), I- and SNAP-2 (>50).

**Table S3. Cosine contents of individual sub-trajectories calculated for the first four principal components.**

| System | PC1 | PC2 | PC3 | PC4 |
| --- | --- | --- | --- | --- |
| Wild | 0.064 | 0.003 | 0.007 | 0.0001 |
| W50S | 0.001 | 0.009 | 0.02 | 0.005 |
| R52C | 0.0004 | 0.013 | 0.015 | 0.005 |
| D104G | 0.0004 | 0.0003 | 0.006 | 0.00007 |

**Table S4.** List of TREM2 variants that have been documented to be associated with neurodegeneration. All common and rare variants and experimental evidence of loss of function are represented along with MD simulation consequences.

| Substitution | Disease  association | Experimental evidence | Number of tools predicted deleterious^a^ | Evidence from molecular dynamics simulation | | | References |
| --- | --- | --- | --- | --- | --- | --- | --- |
|  |  |  |  | Change of dynamical motion | Change of inter-residue contact in CDR binding site | CDR conformational remodeling |  |
| A28V | FTD | N/A | 2 |  |  |  | 1 |
| S31F | FTD | N/A | 11 |  |  |  |  |
| Y38C | NHD ^2^ | LB | 11 | Yes | Yes | Yes | ^3^ , ^4,5^ |
| D39E | AD ^6^ | N/A | 4 |  |  |  |  |
| R47H | AD ^2^ | LB | 8 | Yes | Yes | Yes | 3,7 |
| R47C | FTD | N/A | 10 |  |  |  |  |
| W50C | NHD ^8^ | LB | - | Yes | Yes | Yes | 5,9 |
| C51Y | N/A | N/A | 11 |  |  |  |  |
| R52H | AD ^10^ | N/A | 14 |  |  |  |  |
| R62H | AD ^2^ | LB | 1 | Yes | Yes | Yes | 3,7,9 |
| T66M | NHD ^2^ | LB | 10 | Yes | Yes | Yes | ^3^, ^4,5^ |
| N68K | N/A |  | 4 | Yes | No | Yes | 7 |
| T85K | N/A | N/A | 4 |  |  |  |  |
| D86V | FTD |  | - | Yes | Yes | Yes | 7 |
| D87N | AD ^2^ | LB | 7 |  |  |  | 3 |
| T96K | AD ^2^ | IB | 10 | Yes | Yes | Yes | 2,7 |
| R98W | AD ^11^ | N/A | 9 |  |  |  |  |
| R98Q | N/A | N/A | 5 |  |  |  |  |
| A105V | N/A | N/A | 12 |  |  |  |  |
| V126G | NHD ^2^ | N/A | 15 | Yes | Yes | Yes | 4,5 |
| H157Y | AD | N/A | 5 |  |  |  |  |

N/A: Not available, FTD: Frontotemporal Dementia, NHD: Nasu-Hakola disease, AD: Alzheimer's Disease, MDS: Molecular Dynamics Simulation, LB: Loss of Binding, IB: Increase of Binding. ^a^Deleterious as predicted by the present study.


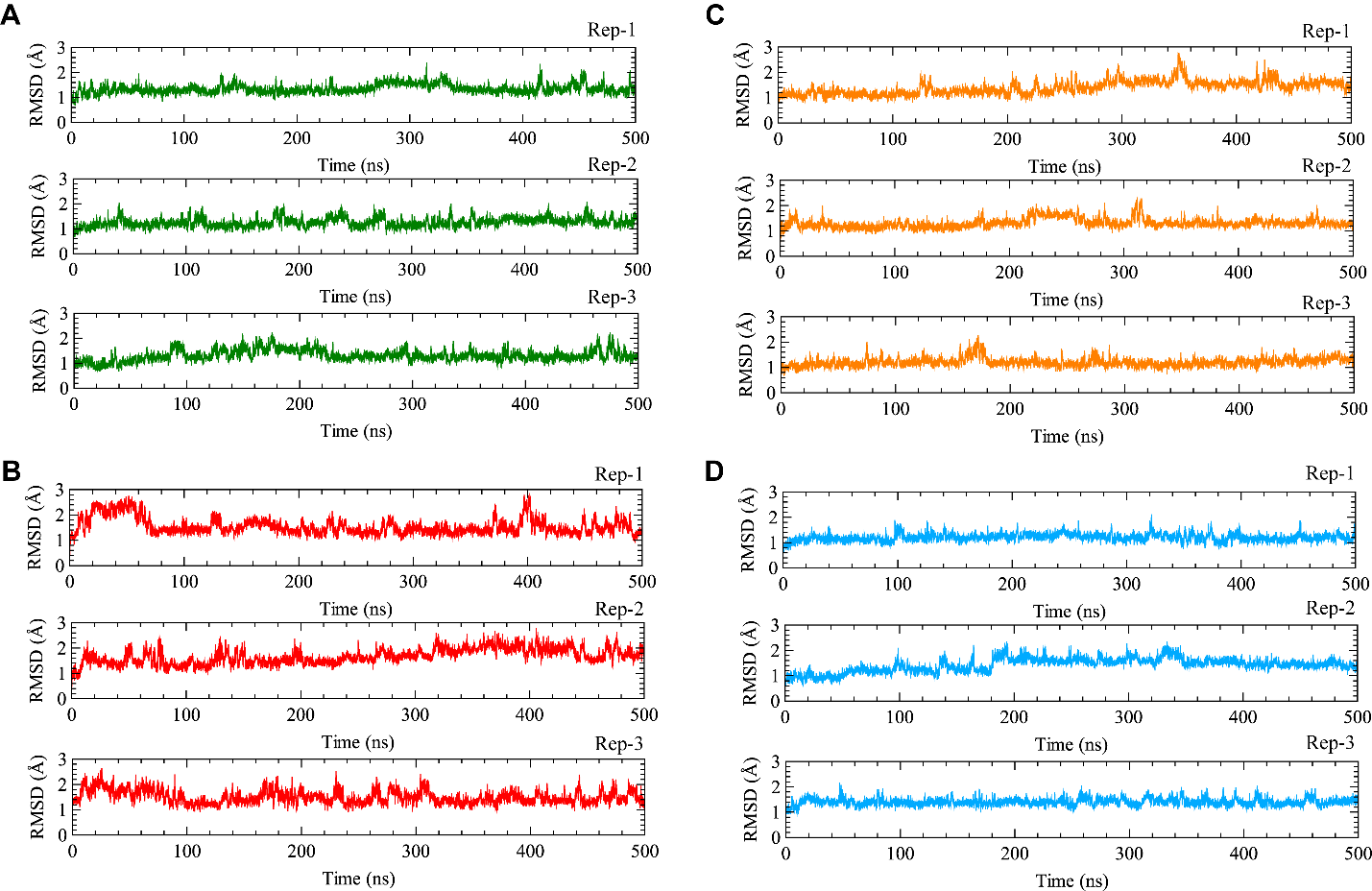


**Figure S1.** RMSD values of wild and variant-containing structures derived from runs of individual systems, calculated based on protein c-alpha of the wild-type (A), W50S (B), R52C (C), and D104G (D).


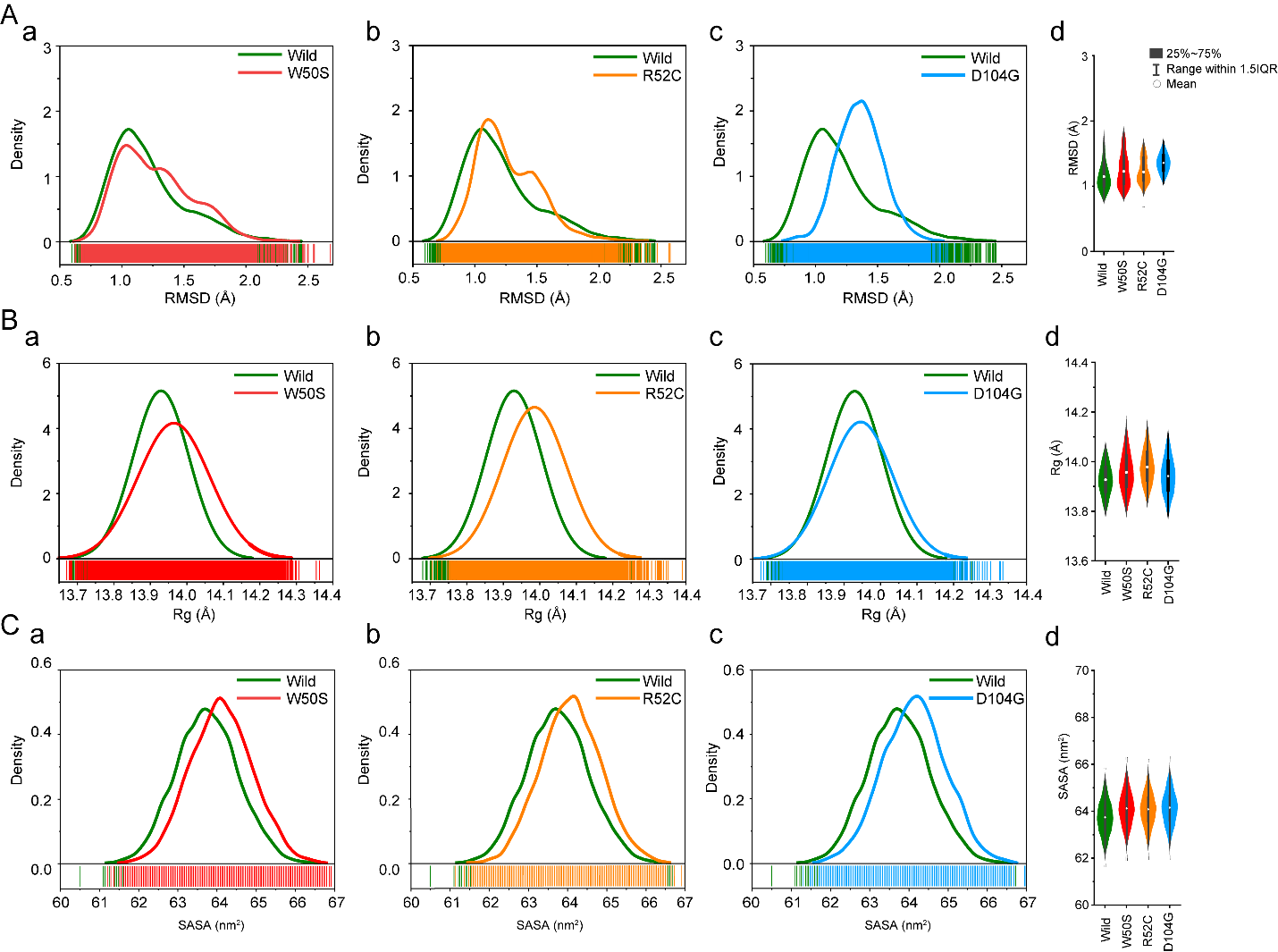


**Figure S2.** Variant-induced changes in the conformational stability of TREM2. Graphical illustrations representing conformational changes of variants versus the wild-type as determined by RMSD (A), Rg (B), and SASA (C) analyses. In all cases, (a) represents the probability density of W50S (a), R52C (b), and D104G (c) versus the wild-type. Mean differences are represented by violin plots.


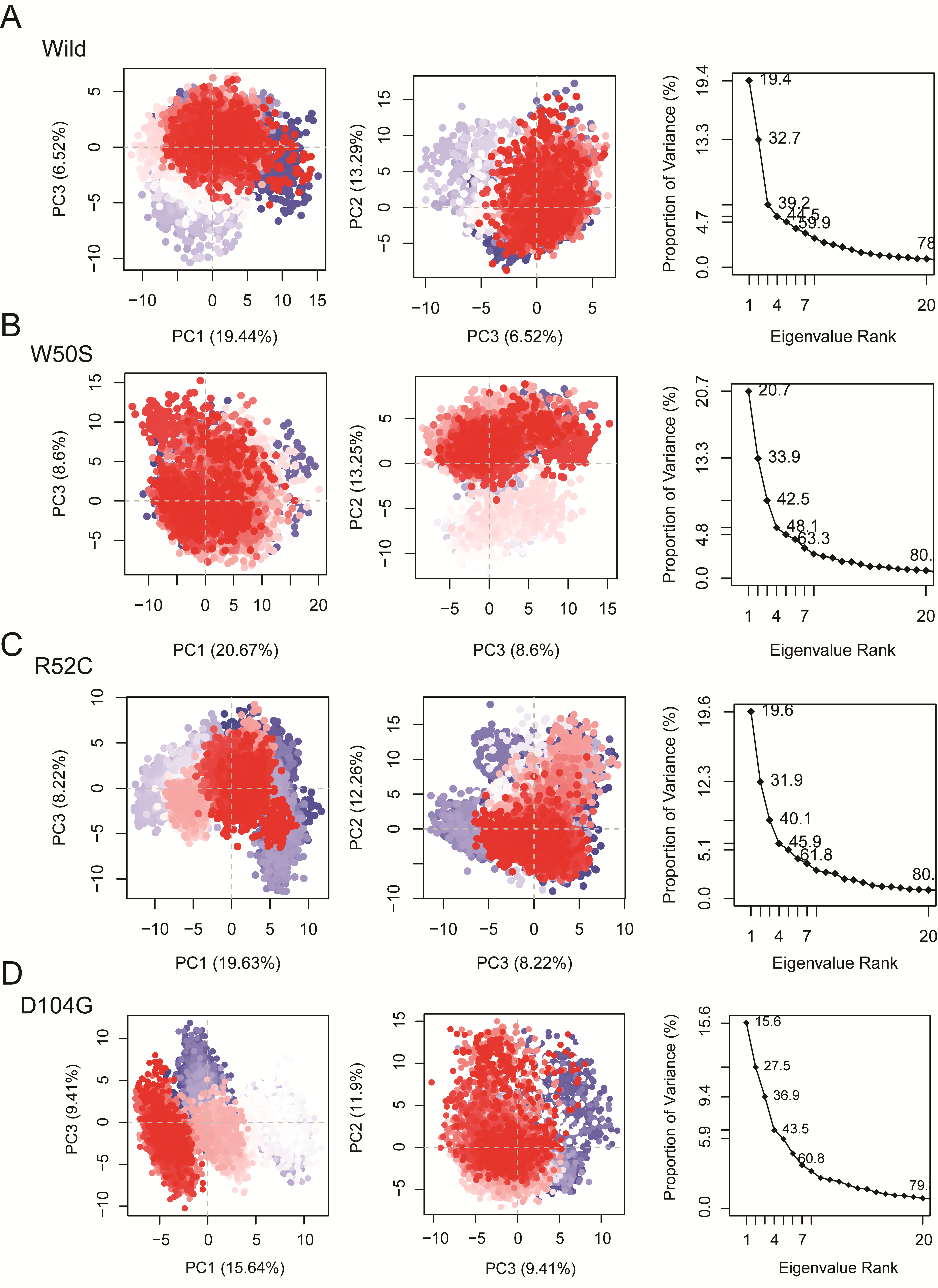


**Figure S3.** Conformational distributions of each protein were captured using the first three principal components. A single protein conformer is represented by a dot, and the simulation is time color-coded from blue to white to red. The proportion of variance captured by eigenvectors is also shown for wild-type (A), W50S (B), R52C (C), and D104G (D).

**
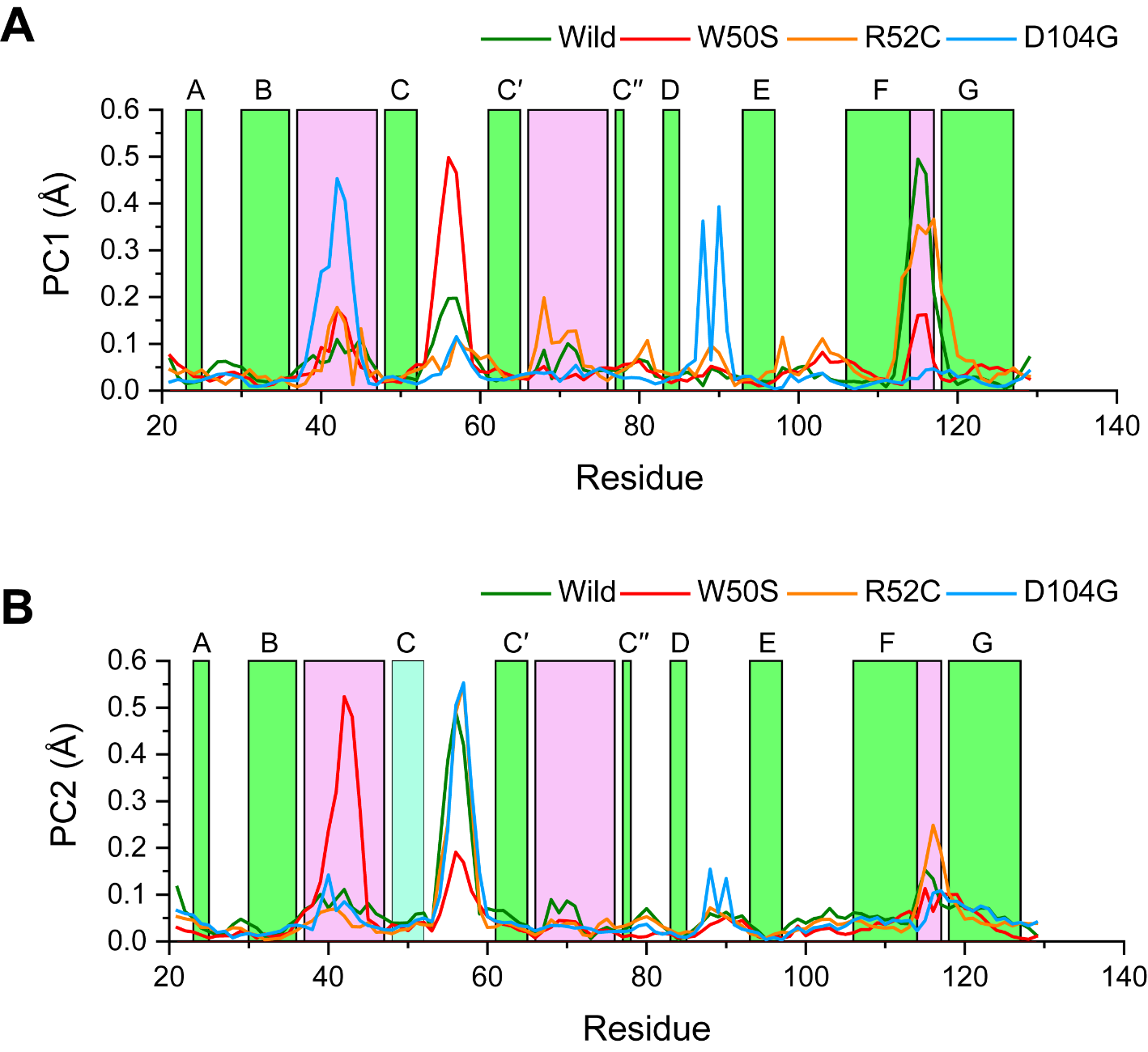
**

**Figure S4.** Line plots showing the degree of mobility represented by PC1 (A) and PC2 (B) values for the three variants and the wild-type.


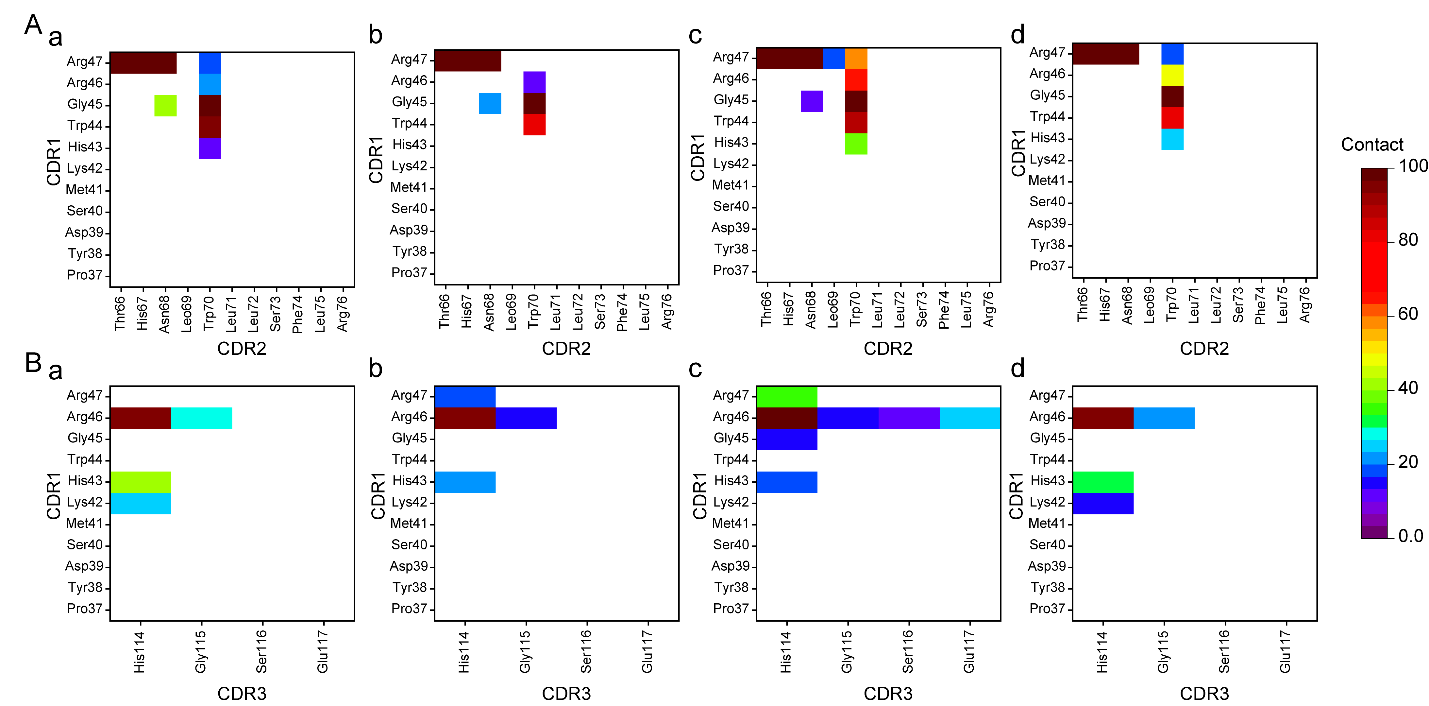


**Figure S5.** Heatmap representing the magnitudes of inter-residue contacts between CDR1 and CDR2 (A) or CDR3 (B). In all case, wild-type (a), W50S (b), R52C (c), and D104G (d). Intermolecular total contact percentages are summarized in the color-coded plots, where the red to blue color scale bar represents higher to lower total contact percentages.


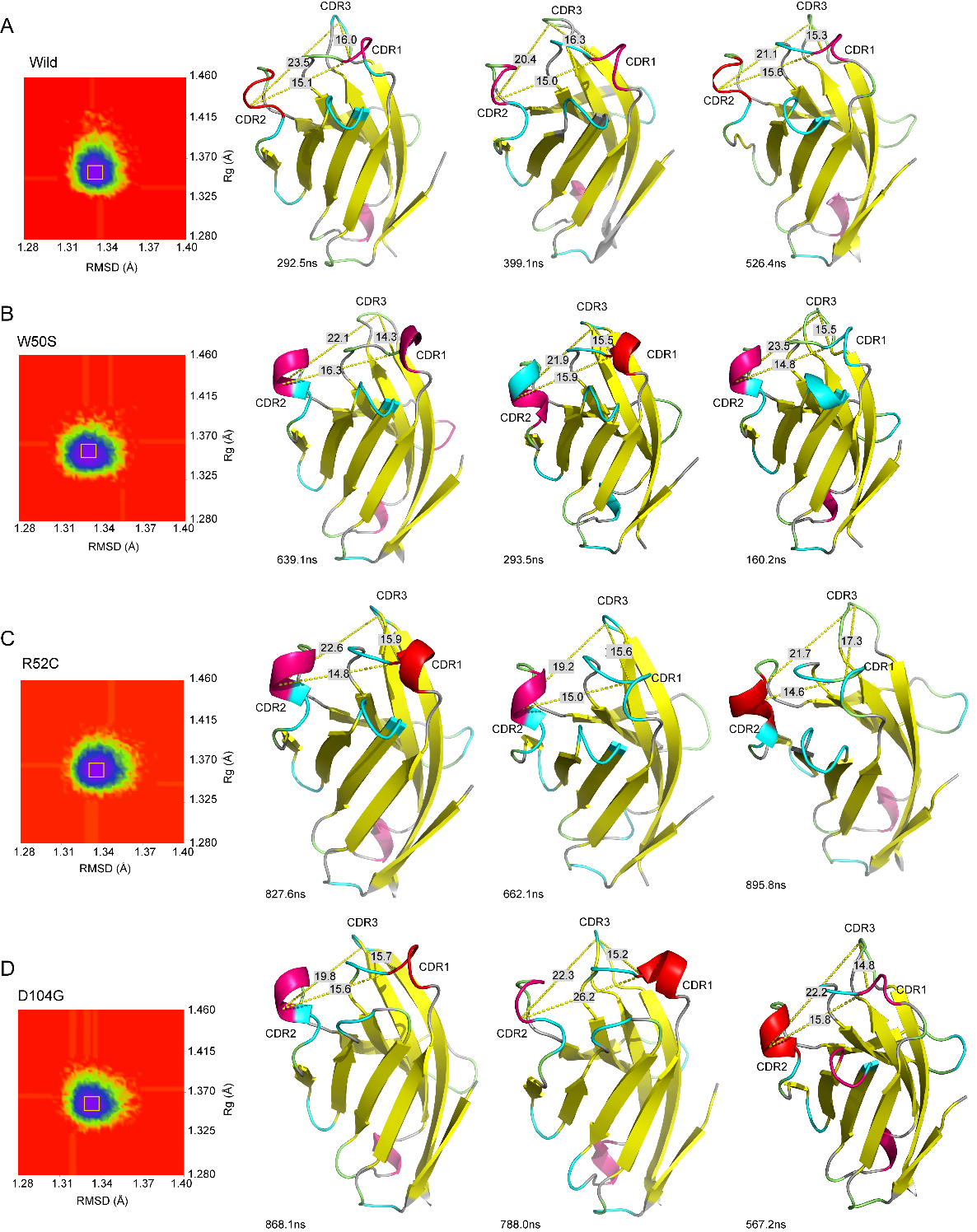


**Figure S6.** Cartoon representations of lowest minimal energy states for wild and variants, retrieved from free energy landscape (FEL), marked by the yellow box in individual 2D FEL plot. (A) wild-type, (B) W50S, (C) R52C, and (D) D104G showing structural organizational differences caused by variants. The wild type exhibited more stable CDR states than the variants. Cartoons are color-coded according to the DSSP classification.

**Supplemental Videos**

**Supplemental Video 1:** A movie showing the trajectories of three independent simulations (each run of 500 ns) for wild-type.

**Supplemental Video 2:** A movie showing the trajectories of three independent simulations (each run of 500 ns) for W50S.

**Supplemental Video 3:** A movie showing the trajectories of three independent simulations (each run of 500 ns) for R52C.

**Supplemental Video 4:** A movie showing the trajectories of three independent simulations (each run of 500 ns) for D104G.

**References**

1 Thelen, M. *et al.* Investigation of the role of rare TREM2 variants in frontotemporal dementia subtypes. *Neurobiology of aging* **35**, 2657. e2613-2657. e2619 (2014).

2 Kober, D. L. *et al.* Neurodegenerative disease mutations in TREM2 reveal a functional surface and distinct loss-of-function mechanisms. *Elife* **5**, e20391 (2016).

3 Yeh, F. L., Wang, Y., Tom, I., Gonzalez, L. C. & Sheng, M. TREM2 binds to apolipoproteins, including APOE and CLU/APOJ, and thereby facilitates uptake of amyloid-beta by microglia. *Neuron* **91**, 328-340 (2016).

4 Dash, R., Choi, H. J. & Moon, I. S. Mechanistic insights into the deleterious role of nasu-hakola disease associated TREM2 variants. *bioRxiv*, 705608 (2019).

5 Dash, R., Choi, H. J. & Moon, I. S. Mechanistic insights into the deleterious roles of Nasu-Hakola disease associated TREM2 variants. *Sci Rep* **10**, 3663, doi:10.1038/s41598-020-60561-x (2020).

6 Cuyvers, E. *et al.* Investigating the role of rare heterozygous TREM2 variants in Alzheimer's disease and frontotemporal dementia. *Neurobiology of aging* **35**, 726. e711-726. e719 (2014).

7 Dean, H. B., Roberson, E. D. & Song, Y. Neurodegenerative Disease-Associated Variants in TREM2 Destabilize the Apical Ligand-Binding Region of the Immunoglobulin Domain. *Frontiers in neurology* **10**, 1252-1252, doi:10.3389/fneur.2019.01252 (2019).

8 Dardiotis, E. *et al.* A novel mutation in TREM2 gene causing Nasu-Hakola disease and review of the literature. *Neurobiology of aging* **53**, 194. e113-194. e122 (2017).

9 Brownjohn, P. W. *et al.* Functional Studies of Missense TREM2 Mutations in Human Stem Cell-Derived Microglia. *Stem Cell Reports* **10**, 1294-1307, doi:10.1016/j.stemcr.2018.03.003 (2018).

10 Jin, S. C. *et al.* Coding variants in TREM2 increase risk for Alzheimer's disease. *Human molecular genetics* **23**, 5838-5846 (2014).

11 Guerreiro, R. *et al.* TREM2 variants in Alzheimer's disease. *New England Journal of Medicine* **368**, 117-127 (2013).
